# Stress‐induced Rab11a‐exosomes induce amphiregulin‐mediated cetuximab resistance in colorectal cancer

**DOI:** 10.1002/jev2.12465

**Published:** 2024-06-18

**Authors:** John D. Mason, Ewan Marks, Shih‐Jung Fan, Kristie McCormick, Clive Wilson, Adrian L. Harris, Freddie C. Hamdy, Chris Cunningham, Deborah C. I. Goberdhan

**Affiliations:** ^1^ Department of Physiology, Anatomy and Genetics University of Oxford Oxford UK; ^2^ Department of Life Sciences National Central University Taoyuan City Taiwan; ^3^ Department of Oncology, Weatherall Institute of Molecular Medicine University of Oxford Oxford UK; ^4^ Nuffield Department of Surgical Sciences University of Oxford, John Radcliffe Hospital Oxford UK

**Keywords:** AREG, cetuximab, colorectal cancer, extracellular vesicle, EGFR, Rab11a‐exosome

## Abstract

Exosomes are secreted vesicles made intracellularly in the endosomal system. We have previously shown that exosomes are not only made in late endosomes, but also in recycling endosomes marked by the monomeric G‐protein Rab11a. These vesicles, termed Rab11a‐exosomes, are preferentially secreted under nutrient stress from several cancer cell types, including HCT116 colorectal cancer (CRC) cells. HCT116 Rab11a‐exosomes have particularly potent signalling activities, some mediated by the epidermal growth factor receptor (EGFR) ligand, amphiregulin (AREG). Mutant activating forms of KRAS, a downstream target of EGFR, are often found in advanced CRC. When absent, monoclonal antibodies, such as cetuximab, which target the EGFR and block the effects of EGFR ligands, such as AREG, can be administered. Patients, however, inevitably develop resistance to cetuximab, either by acquiring KRAS mutations or via non‐genetic microenvironmental changes. Here we show that nutrient stress in several CRC cell lines causes the release of AREG‐carrying Rab11a‐exosomes. We demonstrate that while soluble AREG has no effect, much lower levels of AREG bound to Rab11a‐exosomes from cetuximab‐resistant KRAS‐mutant HCT116 cells, can suppress the effects of cetuximab on KRAS‐wild type Caco‐2 CRC cells. Using neutralising anti‐AREG antibodies and an intracellular EGFR kinase inhibitor, we show that this effect is mediated via AREG activation of EGFR, and not transfer of activated KRAS. Therefore, presentation of AREG on Rab11a‐exosomes affects its ability to compete with cetuximab. We propose that this Rab11a‐exosome‐mediated mechanism contributes to the establishment of resistance in cetuximab‐sensitive cells and may explain why in cetuximab‐resistant tumours only some cells carry mutant KRAS.

## Graphical abstract

This study highlights a clinically relevant mechanism in which stress‐induced Rab11a‐exosomes carrying the EGFR ligand, amphiregulin (AREG) transfer drug resistance between genetically distinct colorectal cancer cells. Resistance to cetuximab, an anti‐EGFR therapy, can be passed via Rab11a‐exosomes from drug‐resistant KRAS‐mutant cells to previously drug‐responsive KRAS‐wild type cells. Unlike soluble AREG, Rab11a‐exosome‐associated AREG competes with cetuximab to activate EGFR signalling and promote EGFR‐dependent outcomes, such as growth. This mechanism may support the co‐operative evolution of clonal heterogeneity during tumour progression.

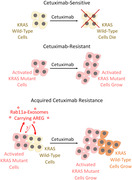

## 1 INTRODUCTION

Extracellular vesicles (EVs) are membrane‐bound structures that appear to be secreted by all cell types (van Niel et al., 2022), and which deliver cargos to recipient cells that play important roles in both physiology and pathology (Cheng & Hill, 2022; Lucotti et al., 2022; Yates et al., 2022). EVs can be produced by outward protrusion and detachment from the plasma membrane, forming so‐called ectosomes, or by inward budding of endosomal membranes to produce vesicles, which are secreted as exosomes, when these endosomes fuse with the plasma membrane (Dixson et al., 2023).

EVs can be separated and concentrated from conditioned medium or biofluids by methodologies based on physical properties, such as size and density, but the resulting preparations contain heterogenous mixtures of exosomes and ectosomes, as well as aggregated proteins (Jeppesen et al., 2019). For example, small EVs (sEVs) separated and concentrated by differential centrifugation (dUC) or size‐exclusion chromatography (SEC) typically contain exosomes, which have a size range of 30–150 nm in diameter, but also include ectosomes of similar size.

Microenvironmental stress suppresses signalling by nutrient‐sensitive mechanistic Target of Rapamycin Complex 1 (mTORC1; reviewed in Goberdhan et al., 2016). We have identified a specific subtype of exosome that is preferentially secreted under such stress conditions by colorectal, prostate and cervical cancer cell lines. This stress‐induced change is characterised by a switch in exosome biogenesis and secretion, involving reduction in classical CD63‐positive exosomes generated in late endosomal compartments and an increase in those formed in Rab11a‐labelled recycling endosomes, termed Rab11a‐exosomes (Fan et al., 2020). These exosomes are also made under normal physiological conditions, and in some cases constitute a high proportion of secreted EVs, for example, in the prostate‐like secondary cells of the fruit fly, *Drosophila melanogaster*, where they mediate reproductive signalling between males and females (Corrigan et al., 2014; reviewed in Wilson et al., 2017).

One major exosome biogenesis mechanism involves the endosomal complexes required for transport (ESCRT) proteins, which load exosome cargos, distort the endosomal compartment membrane during inward budding and ultimately pinch off intraluminal vesicles (ILVs; Vietri et al., 2019). We have recently shown that Rab11a‐, but not late endosomal, exosome formation can be blocked by knockdown of several accessory ESCRT‐III proteins (Marie et al., 2023). These experiments have revealed that Rab11a‐exosomes have unique activities in physiology and disease, for example, promoting growth in the colorectal cancer (CRC) HCT116 cell line via a membrane‐bound form of amphiregulin (AREG), the ligand for the EGF receptor (EGFR, otherwise known as human EGFR‐related 1, HER1) ligand (Marie et al., 2023). This exosome‐associated AREG activates the EGFR at concentrations much lower than soluble AREG (Fan et al., 2020).

In the UK, colorectal cancer (CRC) is the fourth most common cancer, with an incidence of approximately 42,000 new cases and 16,000 mortalities per year (Cancer Research UK, 2023). Survival is closely related to disease stage at diagnosis, ranging from 95% five‐year survival for stage I to below 10% for stage IV. Surgical resection with or without neoadjuvant or adjuvant chemotherapy (or chemoradiotherapy) forms the basis of treatment with curative intent.

One important form of chemotherapy involves cetuximab, and related chimeric monoclonal antibodies, which target the EGFR and block its signalling activity (Fornasier et al., 2018). Approximately 40% of CRC patients carry activating KRAS mutations, and 3% have NRAS mutations, rendering anti‐EGFR therapies ineffective (Bando et al., 2023). Cetuximab can, however, be used in combination treatment of metastatic CRC in patients lacking such downstream mutations, but even in these cases, it is not always effective (Allegra et al., 2016). Eventually all patients develop cetuximab resistance. In some cases, the clonal amplification of rare cells carrying activating KRAS mutations may be responsible or *de novo* KRAS mutations may emerge (Misale et al., 2012). In others, non‐genetic changes in the tumour microenvironment and intercellular communication are involved (Woolston et al., 2019). Indeed, even when KRAS‐mutant cells are present, they often represent a small proportion of the tumour (Misale et al., 2012). It has been proposed that they may release increased levels of EGFR ligands, such as AREG and TGF‐α, which promote resistance in neighbouring KRAS‐wild type cells (Hobor et al., 2014). A better understanding of these complex resistance mechanisms to improve patient outcome is an area of particular interest.

Here we investigate stress‐induced Rab11a‐exosome secretion from several different CRC cell lines and test whether this exosome sub‐type can mediate cetuximab resistance. We show that membrane‐bound AREG located on Rab11a‐exosomes from nutrient‐stressed, KRAS‐mutant HCT116 cells can compete with cetuximab and induce resistance in cells that do not carry KRAS mutations, even when much higher concentrations of soluble AREG have no effect. Our work suggests a novel mechanism by which resistance to anti‐EGFR therapy can be established, producing the heterogeneous clonal populations that are frequently observed in drug‐resistant patients.

## 2 MATERIALS AND METHODS

### 2.1 Cell culture

The CRC cell lines were purchased from ATCC to ensure authenticity. HCT116 cells were cultured in McCoy's 5A medium (Gibco Life Technologies), Caco‐2 cells in EMEM (Gibco Life Technologies) and SW480 and SW620 cells in DMEM GlutaMAX (Gibco Life Technologies). During normal cell culture, all media was supplemented with heat inactivated foetal calf serum (FBS, 10% McCoy's 5A and DMEM GlutaMAX; 20% EMEM) and 1000 U/mL (1%) penicillin‐streptomycin (Gibco Life Technologies). Cells were incubated at 37°C with 5% CO_2_ and were not used beyond passage 20.

### 2.2 EV isolation

Cells were plated at a density of 9 × 10^6^ cells per plate and cultured until they reached 80% confluency. Following this, the media was changed to serum‐free basal medium (DMEM‐F12, Gibco Life Technologies) supplemented with 1% insulin‐transferrin‐selenium solution (ITS, #4140045, Gibco Life Technologies) for 24 h. For glutamine‐depletion isolations, HCT116 cells were grown for 24 h in DMEM‐F12 without L‐glutamine (#21331046 Gibco Life Technologies) supplemented with physiological (2 mM) or low (0.15 mM) glutamine (Gibco Life Technologies). The low dose of glutamine was determined by undertaking dose–response experiments with reducing glutamine concentrations to determine a concentration that significantly decreases 4E‐BP1 hyperphosphorylation (Fan et al., 2020). Torin1 was added at 100 nM for SW480 cells and 150 nM for Caco‐2 and SW620 cells.

Following 24 h of cell growth in the relevant isolation conditions, the medium was harvested at pre‐cleared by centrifuging at 500 × *g* and 2000 × *g* both for 10 min at 4°C to remove cells, non‐vesicular debris and larger vesicles. Following this, the supernatant was filtered using 0.22 μm vacuum filters (Milex). The filtrate was then concentrated using a tangential flow filter with a 100 kDa membrane (Viva‐flow 50R, Sartorius), followed by ultrafiltration using 100 kDa Amicon Ultra‐15 units (Milex) at 4000 × *g* for 5–10 min at 4°C to a final volume of 1 mL. EVs were then isolated using size‐exclusion chromatography, by injecting the sample onto a 24 × 1 cm size exclusion column containing Sepharose 4B Fast Flow resin (pore size 84 nm) set‐up in an AKTA Start System (GE Healthcare Life Science). EVs were eluted in PBS, collecting 30 × 1 mL fractions. Fractions corresponding to the initial EV peak were pooled into 100‐kDa Amicon Ultra‐4 tubes and centrifuged at 4000 × *g* at 4°C to a final volume of approximately 120 μm for analysis. EVs were stored for a maximum of 24 h at 4°C for transmission electron microscopy or functional analysis, or long term at −80°C for nanoparticle tracker analysis or western blotting.

### 2.3 Transmission electron microscopy

Approximately 0.2 μg/μL of purified EVs were incubated on glow discharged 300‐mesh 3 mm carbon‐coated copper grids (TAAB Laboratories) for 2 min and then blotted dry and negatively stained with 2% uranyl acetate for 10 s. Following this the grid was blotted and allowed to air dry before imaging on a FEI Tecnai T12 transmission electron microscope (FEI UK Ltd) operated at 120 kV with a Gatan Oneview digital camcer (Gatan Inc) at 120,000× magnification.

### 2.4 Nanoparticle tracker analysis

The NS500 NanoSight^®^ was used to capture five 30 s videos per EV sample (diluted 1:500 in PBS). Particle movement was assessed using NTA software 3.2 (NanoSight Ltd) to generate graphs plotting EV concentration (×10^8^ mL^−1^) against particle size (nm).

### 2.5 Western blotting

Cell lysates were collected by lysing cells in RIPA buffer supplemented with protease and phosphatase inhibitors (Sigma) and centrifugation at 14,000 rpm for ten min at 4°C. Total protein context was measured using a BCA protein assay kit (Pierce, Thermo Scientific). When undertaking western blotting on EVs, we loaded gels with EV preparations from the same protein mass of secreting cells so that changes in band intensity on the blots with glutamine depletion or Torin1 exposure reflected a net change in secretion of the marker on a per cell basis. We have previously shown that the normalisation method used during western analysis does not affect the overall conclusions made (Fan et al., 2020).

Equal amounts of protein or EVs were mixed with reduced (containing 5% β‐mercaptoethanol, Sigma) or non‐reduced 4 x Laemmli sample buffer (Bio‐Rad) and denatured at 95°C for 10 min. Samples were then electrophoretically separated on 10% mini‐PROTEAN^®^ precast gels (Bio‐Rad), followed by wet‐transfer onto a Immobilin^®^‐Fl polyvinylidene difuoride membrane with a 0.22 μm pore size (Merck) at 100 V for one hour at 4°C. Membranes were then blocked in 5% bovine serum albumin (BSA, Sigma) for reduced conditions or 5% semi‐skimmed milk (Sigma) for non‐reduced conditions, both in TBS containing 1% Tween‐20 (TBST, Sigma) for one hour at room temperature. Membranes where then incubated with primary antibodies diluted in the same blocking buffer overnight at 4°C. The following day, membranes underwent 3 × 10‐min washes in TBST and then probed with the relevant secondary HRP‐conjugated antibody. The membranes then underwent a further 3 × 20‐min washes in TBST before incubation in Trident femto Western HRP solution (GeneTex) and visualisation using the ChemiDoc Touch Imaging System (Bio‐Rad). Relative band intensities were quantified using ImageJ and normalised to tubulin or cell lysate levels.

Antibody suppliers, catalogue numbers of concentrations used were as follows: rabbit anti‐4E‐BP1 (Cell Signalling Technology #9644), rabbit anti‐S6 (Cell Signalling Technology #2217, 1:4000), rabbit anti‐p‐S6‐Ser240/244 S6 (Cell Signalling Technology #5364, 1:4000), rabbit anti‐Akt (Cell Signalling #9272, 1:1000), rabbit anti‐p‐Akt‐Ser473 (Cell Signalling #4060, 1:500), rabbit anti‐Caveolin‐1 (Cell Signalling Technology #3238, 1:500), goat anti‐AREG (R&D Systems #AF262, 1:200), mouse anti‐Tubulin (Sigma #T8328, 1:4000), mouse anti‐CD81 (Santa Cruz #23962, 1:500), mouse anti‐CD63 (BD Biosciences # 556019, 1:500), mouse anti‐Alix (Abcam ab117600, 1:500), rabbit anti‐Syntenin‐1 antibody (Abcam ab133267, 1:500), rabbit anti‐Tsg101 (Abcam ab125011, 1:500), mouse anti‐Rab11 (BD Biosciences #610657, 1:500), rabbit anti‐EGFR (Cell Signalling #4267, 1:1000), rabbit anti‐p‐EGFR‐Tyr1068 (Cell Signalling #3777, 1:500), rabbit anti‐p44/42 MAPK (ERK; Cell Signalling Technology #4695, 1:1000), rabbit anti‐p‐p44/42 MAPK (Cell Signalling Technology #4370, 1:1000), rabbit anti‐EEA‐1 (Cell Signalling Technology #3288, 1:500), sheep anti‐TGN46 (Bio‐Rad; AHP500G, 1:1,000), rabbit anti‐Calnexin (Abcam #ab213243, 1:1,000), anti‐mouse IgG (H+L) HRP conjugate (Promega #W4021, 1:10000), anti‐rabbit IgG (H+L) HRP conjugate (Promega #W4011, 1:10000), anti‐goat IgG (H+L) HRP conjugate (R&D Systems #HAF109, 1:100).

### 2.6 Growth assays

Recipient cells were seeded in 96‐well plates (HCT116 cells at 2 × 10^3^, SW480 and Caco‐2 cells at 3 × 10^3^ and SW620 cells 4 × 10^3^ per well) in 200 μL of medium following pre‐treatment with freshly prepared EVs (4 × 10^3^ per cell) for 30 min at 37°C. Growth assays were undertaken in reduced serum conditions (HCT116 cells, 1% FBS, SW480, SW620 and Caco‐2 cells 0.25% FBS) and measured over five days by live image acquisition using the IncuCyte ZOOM^®^ Live Cell Imager (Essen Bioscience). The IncuCyte analysis software was used to automatically detect cell edges in order to create a confluency mask, which was used to calculate cellular growth and normalised to confluence at time zero, giving a fold change in confluency. Eight technical repeats were employed for each condition and each experiment was repeated at least three times unless otherwise stated.

AREG‐neutralising experiments were performed by pre‐incubating freshly prepared EVs (at 4 × 10^3^ EVs per recipient cell) with either 4 μg/mL of goat anti‐AREG (R&D Systems #AF262) or goat anti‐IgG (R&D Systems #AB‐1080‐C) as a control antibody in the relevant reduced serum media, at 37°C for 2 h (Fan et al., 2020). Following this, the EVs were added to the recipient cells and analysed as described above. Experiments investigating EV‐free AREG were undertaken by incubating recipient cells with 0.03–3000 ng/mL recombinant human Amphiregulin protein (R&D Systems #262‐AR‐100), for 30 min at 37°C before plating as described above.

Drug and inhibitor experiments were undertaken by adding the drug (research grade cetuximab 30 μg/mL, R&D Systems MAB9577) or inhibitor (AG1478 hydrochloride 2.5 mM, Tocris #1276, BMS599626 dihydrochloride 2.5 mM, Tocris #5022) to the recipient cells at the same time as the freshly prepared EVs and incubated for 30 min at 37°C before plating.

### 2.7 Assessment of EV‐induced pEGFR and pERK activation

HCT116 or Caco‐2 cells were plated in 6‐well plates in full FBS supplemented media at a density of 4 × 10^5^ cells per well. After 24 h the cells were serum starved (0% FBS) overnight and the following day freshly prepared EVs (4 × 10^3^ per cell) were pre‐incubated with or without AREG‐neutralising antibody (4 μg/mL) for 2 h at 37°C and added to the recipient cells in fresh 0% FBS media. Cell lysates were then collected after 5 min for EGFR analysis and 15 min for ERK activation for analysis by Western blotting.

### 2.8 Statistical analysis

For Western blot analysis, relative signal intensities were analysed using the Kruskal–Wallis or Mann–Whitney *U* test. For growth assays, data was analysed by two‐way Analysis of Variance (ANOVA) with a Tukey post hoc test for multiple comparisons. All data is presented as mean ± standard deviation and a statistically significant result was defined as *p* < 0.05. Data analysis was performed using GraphPad Prism version 8.3.1 (GraphPad Software, Inc).

## 3 RESULTS

### 3.1 Rab11a‐exosome secretion from a range of CRC cell lines is increased in response to mTORC1 inhibition

**FIGURE 1:**
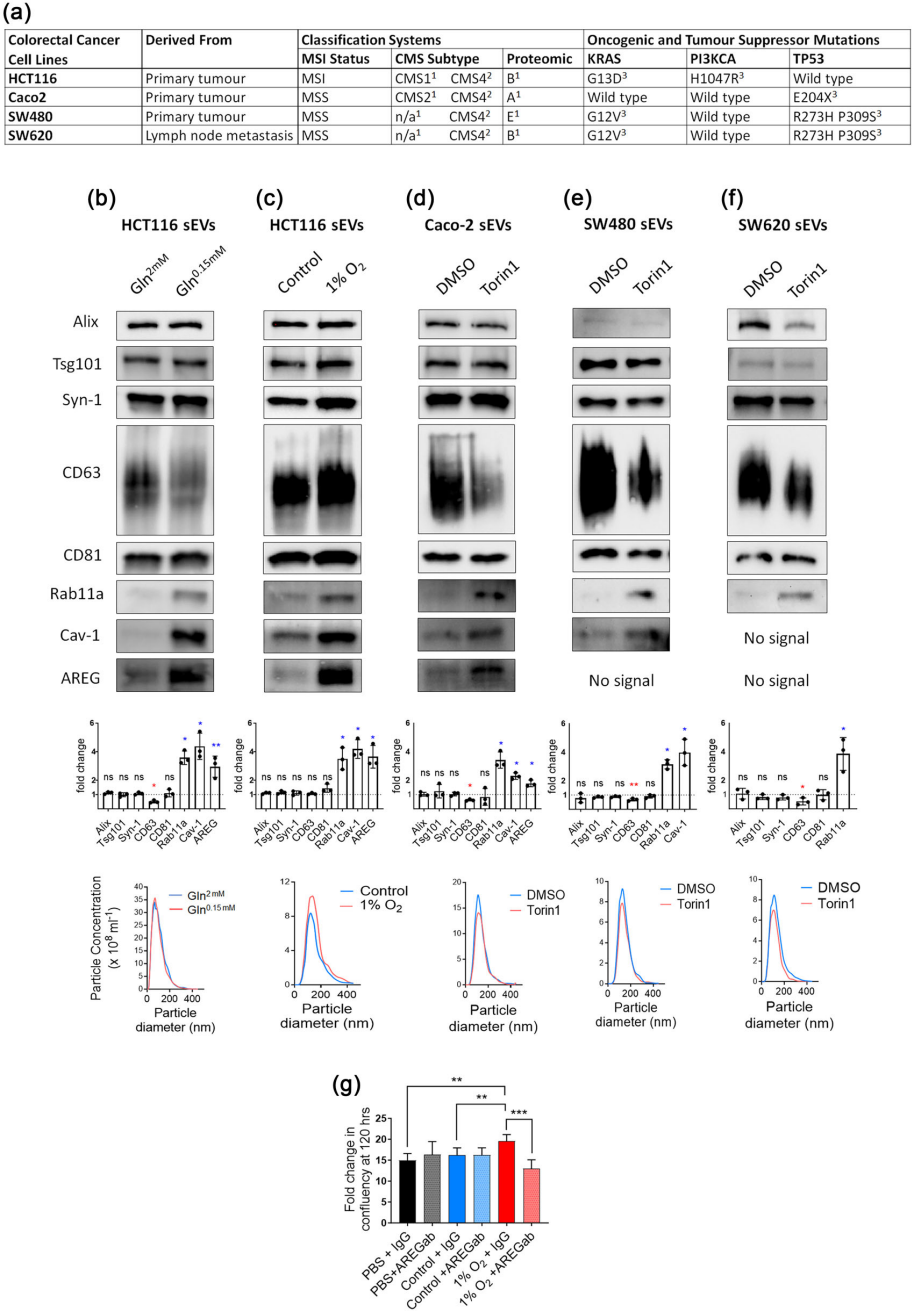
Inhibition of mTORC1 induces secretion of Rab11a‐exosomes in multiple CRC cell lines. (a) Classification of the four CRC cell lines investigated based on molecular pathways and mutational status in critical oncogenes and tumour suppressor genes. (b) Western blot analysis of sEVs isolated from HCT116 cells cultured in glutamine‐depleted (Gln^0.15 mM^) versus control glutamine‐replete (Gln^2 mM^) conditions. Note characteristic increase in Rab11a, AREG and Cav‐1 levels and the reduction in CD63 that are consistently observed (see histogram, where changes in protein levels, measured by densitometry and normalised to protein levels in secreting cell lysates, are plotted for three independent experiments; mean ± SD). NTA analysis reveals that the total number and size distribution of secreted particles is unchanged by this treatment. (c) Western blot of sEV proteins isolated from HCT116 cells cultured in hypoxic (1% O_2_) versus normoxic conditions also reveals an increase in markers of Rab11a‐exosomes (Rab11a and AREG) and other stress‐induced sEVs (Cav‐1). (d–f) Western blots of sEV proteins from Caco‐2 (d), SW480 (e) and SW620 (f) cells treated with the ATP‐competitive mTORC1 inhibitor, Torin1 (100 nM for SW480 cells and 150 nM for Caco‐2 and SW620 cells), versus treatment with vehicle alone (DMSO) also reveals a switch to secretion of increased Rab11a and AREG, and a reduction in CD63. NTA reveals that all sEV preparation have similar size distribution, but particle numbers were generally slightly reduced, ie. SW480: 41.58 ± 0.73 × 10^8^ particles/mL (Torin1) versus 42.58 ± 1.74 × 10^8^ (control) particles/mL: SW620: 13.71 ± 0.47 × 10^8^ particles/mL (Torin1) versus 15.20 ± 0.37 × 10^8^ (control) particles/mL: Caco2: 14.37 ± 0.98 × 10^8^ particles/mL (Torin1) versus 18.11 ± 1.27 × 10^8^ (control) particles/mL. (g) Graph showing levels of HCT116 growth after 120 h, measured as fold change in confluency, following addition of PBS, control HCT116 sEVs, or hypoxia‐induced HCT116 sEVs in the presence or absence of pre‐incubation with neutralising anti‐AREG antibodies. Note that only sEVs isolated under hypoxic conditions enhance growth and this is AREG‐dependent. *P < 0.05. Red and blue asterisks denote reduction and increase in protein levels respectively in Rab11a‐exosome‐enriched sEVs. MSI, microsatellite instability; MSS, microsatellite stability; CMS, consensus molecular subtype; n/a, not applicable. KRAS activating mutations: G13D or G12V. PI3K activating mutations: E545K D549N or H1047R. TP53 inactivating mutations: R273H P309S, or S241F, or E204X. Superscripts 1,2,3 refer to ^1^Ahmed et al. (2013); ^2^Sveen et al. (2018); ^3^Wang et al. (2017).

A panel of four cell lines (Figure 1a) representing some of the molecular, cellular and genetic heterogeneity observed in CRC, namely microsatellite instability (MSI)/stability (MSS) (Flecchia et al., 2022); consensus molecular subtype (CMS) (Guinney et al., 2015; Sveen et al., 2018); proteomic signature (Wang et al., 2017); and oncogene/tumour suppressor status (Ahmed et al., 2013), was investigated to determine whether these cells all secrete more Rab11a‐exosomes under nutrient stress. Cells were cultured in serum‐free conditions, but the medium was supplemented with insulin, selenium and transferrin (ITS) to maintain growth factor signalling. Size‐exclusion chromatography (SEC) was employed to separate and concentrate the secreted sEVs, which lacked membrane‐associated markers from the ER, Golgi and early endosomes that should not be excluded from concentrated sEV preparations (Figure S1; Welsh et al., 2024).

In HCT116 cells, downregulation of the mTORC1 signalling pathway in response to glutamine depletion, confirmed by western blot analysis of mTORC1 targets, 4E‐BP1 and S6 (Figure S2a, b), led to a switch to increased secretion of Rab11a‐exosomes carrying membrane‐bound AREG (Figure 1b; cf. Fan et al., 2020; Marie et al., 2023). As previously shown, there was also an increase in sEV‐associated levels of the scaffolding protein Caveolin‐1 (Cav‐1). Experiments using immuno‐affinity‐separation of sEVs and selective inhibition of Rab11a‐exosome secretion suggest that Cav‐1, unlike AREG, is associated with alternative stress‐induced vesicles that co‐separate with Rab11a‐exosomes (Fan et al., 2020; Marie et al., 2023). Consistent with previous studies, glutamine depletion did not affect the expression levels of other exosome and EV proteins in cells, except for CD63, which was reduced under stress conditions in both sEVs (Figure 1b) and cells (Figure S2b). The decrease in CD63 on sEVs is partially caused by a stress‐induced reduction in trafficking and exosome secretion through the late endosomal pathway (Fan et al., 2020). Furthermore, as previously shown, there was no detectable change in the total number or the size range of these sEVs, as determined by Nanoparticle Tracking Analysis (NTA; Figure 1b). Analysis of SEC‐separated sEVs by transmission electron microscopy (TEM) revealed the standard cup‐like morphology typically associated with sEV preparations (Figure S2g).

Hypoxia, a common microenvironmental stress in fast‐growing tumours, previously shown to alter sEV cargos in glioblastoma cells (Kurcharzewska et al., 2013), also inhibited mTORC1, as determined by reduced phospho‐S6 and phosphorylated forms of 4E‐BP1 in western blots of cell lysates (Figure S2c). While only the exosome and sEV proteins CD81 (decreased) and AREG (increased) were significantly changed in cell lysates (Figure S2c), hypoxia induced a significant increase in Rab11a, Cav‐1 and AREG in sEV preparations, consistent with elevated Rab11a‐exosome secretion (Figure 1c). In contrast to glutamine depletion, the levels of sEV‐associated CD63 were not reduced under hypoxia, suggesting that secretion of late endosomal exosomes was not reduced by this treatment. Hypoxia‐induced vesicles preferentially promoted growth of HCT116 cells under serum‐depleted conditions, an activity that could be blocked by adding neutralising anti‐AREG antibodies to the sEV preparations (Figure 1g). A similar inhibitory effect was previously observed on Rab11a‐exosome‐enriched sEV preparations from glutamine‐depleted HCT116 cells (Fan et al., 2020) and suggests that the enhanced growth‐promoting effects are mediated by membrane‐associated AREG loaded on to hypoxia‐induced Rab11a‐exosomes.

For Caco‐2, SW480 and SW620 CRC cell lines, glutamine depletion did not affect mTORC1 activity in a dose‐response experiment, as determined by the phosphorylation state of downstream target 4E‐BP1, and only variably reduced S6 phosphorylation (Figure S3a–d). Each of these cell lines was, however, sensitive to the mTORC1 inhibitor Torin1 in the dose range of between 100–150 nM, which affected both S6 and 4E‐BP1 (Figure S3e–g). Under these conditions, Torin1 treatment induced an increase in sEV‐associated Rab11a and Cav‐1 (although not for SW620 cells, which lack Cav‐1), and a decrease in the tetraspanin CD63 (Figure 1d–f), but without a change in levels of these proteins in cell lysates (except for reduced CD63 levels in Caco‐2 cells; Figure S2d–f). Only sEVs from Caco‐2 cells contained detectable levels of AREG, but as with HCT116 cells, these levels were strongly increased following mTORC1 inhibition. In Caco‐2 cells, however, cellular levels of AREG were not affected by this treatment. Other EV markers, such as Alix, Tsg101, Syn‐1 and CD81, were unchanged in sEV preparations from all three cell types (Figure 1d–f) and also in the secreting cells (Figure S2d–f). For all three cell lines, Torin1 treatment did not significantly alter the size of vesicles in sEV preparations: Caco‐2, 139 ± 58 nm diameter (control) versus 143 ± 57 nm (Torin1); SW480, 149 ± 55 nm (control) versus 150 ± 49 nm (Torin1); SW620, 111 ± 54 nm (control) versus 109 ± 54 (Torin1). sEV secretion, however, was reduced by up to 20% for each of these cell lines, as determined by NTA (Figure 1d–f). EVs from each cell line produced cup‐like particles typical of sEV morphology in TEM studies (Figure S2h–j).

In summary, CRC cell lines with different molecular, cellular and genetic properties can be induced to release increased levels of Rab11a‐exosomes by reducing cellular mTORC1 signalling, suggesting that secretion of these alternative sEVs is a typical response to stresses that suppress this nutrient sensor. Furthermore, for CRC cells in which exosomal AREG can be detected, namely HCT116 and Caco‐2, a membrane‐associated form of AREG is also preferentially secreted under these stress conditions.

### 3.2 HCT116‐ and Caco‐2‐derived Rab11a‐exosome preparations can induce AREG‐dependent growth in Caco‐2 cells

**FIGURE 2:**
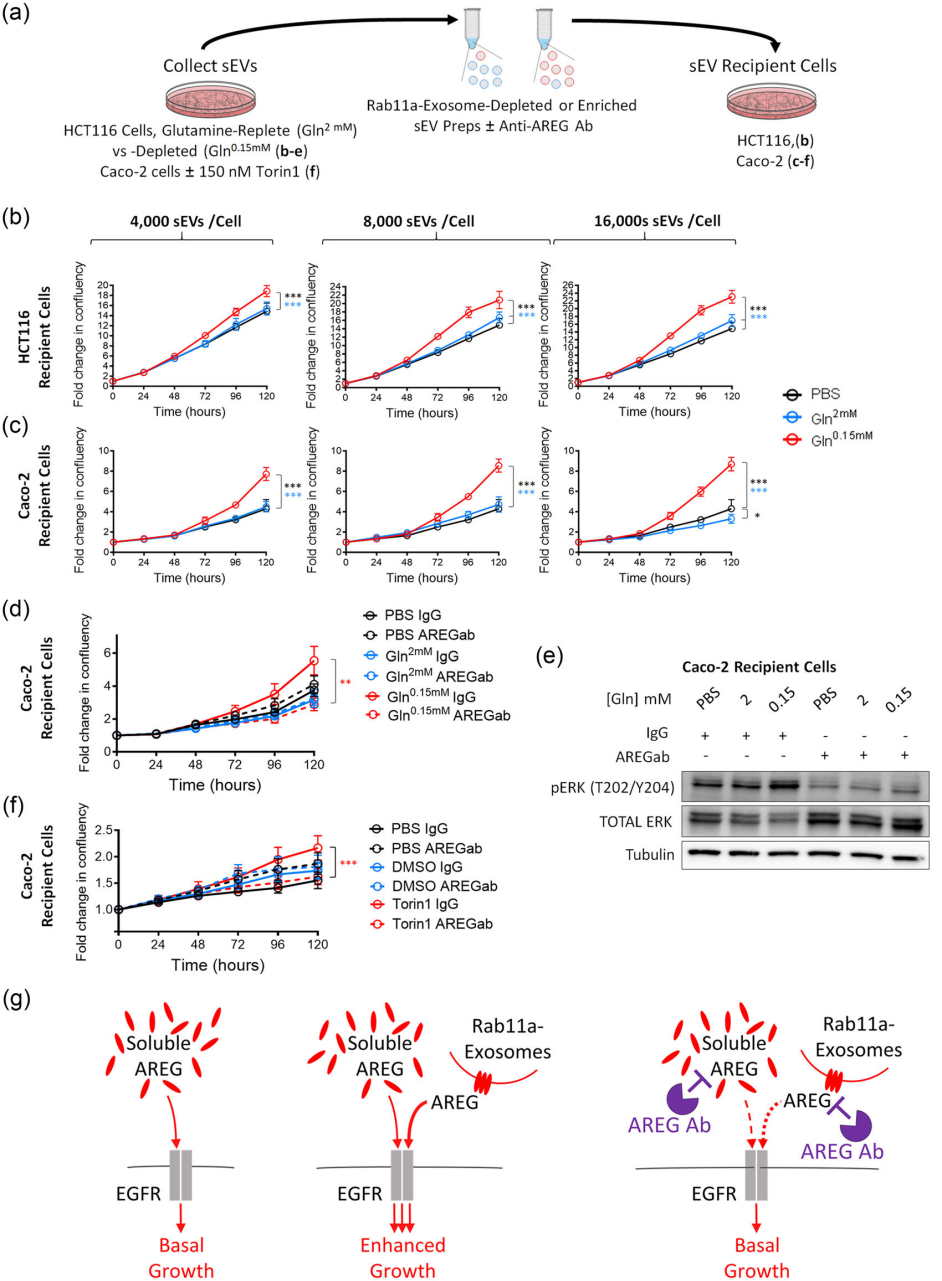
Rab11a‐exosome preparations from HCT116 and Caco‐2 cells enhance growth of HCT116 and Caco‐2 cells in an AREG‐dependent fashion. To test the effect of Rab11a‐exosome‐enriched sEV preparations (red lines on graphs) versus control sEV preparations (blue lines on graphs) on cell growth, sEVs were isolated either from HCT116 cultured in glutamine‐depleted (Gln^0.15 mM^) versus glutamine replete (Gln^2 mM^) conditions; or from Caco‐2 cells cultured in 150 nM Torin1 versus vehicle and compared to PBS addition alone (black line on graphs). They were then added to HCT116 or Caco‐2 recipient cells in reduced serum conditions (1% and 0.25% FBS respectively). (a) Diagram of experimental design. (b) Compared to control sEV preparations (blue line), Rab11a‐exosome‐enriched sEV preparations (red line) promote the growth of HCT116 recipient cells in a dose‐dependent manner (see Fig. S5b). (c) These preparations were also able to promote the growth of Caco‐2 recipient cells, but even the lowest dose of sEVs appeared to produce near‐maximal stimulation (see Fig. S5c). (d) The additional Caco‐2 cell growth stimulated by Rab11a‐exosome‐enriched sEVs isolated from HCT116 cells is blocked by pre‐incubation with an anti‐AREG antibody, which does not significantly affect growth under other conditions. (e) The anti‐AREG antibody blocks the increase in levels of activated phospho‐ERK (P‐ERK) produced in Caco‐2 cells treated with HCT116 Rab11a‐exosome preparations. (f) The additional Caco‐2 cell growth stimulated by Rab11a‐exosome‐enriched sEVs isolated from Caco‐2 cells (red solid line) is blocked by pre‐incubation with an anti‐AREG antibody (red dashed line), which does not significantly affect growth under other conditions. (g) Schematic showing the enhanced growth‐promoting effect of Rab11a‐exosomes, carrying the EGFR ligand AREG (middle), compared to free AREG in serum (left). The additional Rab11a‐exosome‐induced growth is blocked by addition of an anti‐AREG antibody (right). Eight technical repeats were employed for each condition and each experiment was repeated three times. **P < 0.01; ***P < 0.001. Black, blue and red asterisks denote significantly different from PBS control, control sEVs and anti‐AREG antibody treated sEVs respectively.

Rab11a‐exosome preparations isolated from HCT116 cells under glutamine‐depleted conditions can selectively induce AREG‐dependent growth of naïve HCT116 cells cultured in low (1%) serum (Fan et al., 2020; Marie et al., 2023). We confirmed these findings using a range of sEV concentrations (Figure 2a,b and Fig. S4). As previously found, sEVs isolated from glutamine‐replete cells had no detectable growth‐promoting activity. The Rab11a‐exosome preparations stimulated growth in a dose‐dependent fashion (Figure S5a,b), again consistent with our previous observations (Fan et al., 2020).

HCT116 cells carry activating mutations in KRAS and PI3KCA, which regulate two major signalling pathways downstream of the EGFR, making them resistant to EGFR inhibition. Nevertheless, CRC tumours are typically heterogenous, consisting of multiple genetically different clones, some of which carry wild type forms of these oncogenes, even within tumours that are resistant to EGFR‐targeting chemotherapies (Misale et al., 2012). We tested whether the growth‐promoting activity of HCT116 Rab11a‐exosome preparations could also stimulate the growth of cells that did not carry these mutations. Indeed, these preparations selectively promoted the growth of Caco‐2 cells, which lack these mutations, under reduced (0.25%) serum conditions (Figure 2c). These cells were relatively sensitive to Rab11a‐exosome preparations; adding 16 × 10^3^ versus 4 × 10^3^ sEVs per target cell produced a small, but non‐significant, increase in growth (Figure S5c). Consistent with previous experiments using HCT116 Rab11a‐exosome preparations and HCT116 target cells (Fan et al., 2020) and with other cancer sEVs (Raimondo et al., 2019), this effect was blocked by a neutralising anti‐AREG antibody (Figure 2d), which suppressed the sEV‐induced activation via phosphorylation of the downstream signalling target ERK (Figure 2e,g).

Since mTORC1‐inhibited Caco‐2 cells also secrete increased levels of Rab11a‐exosomes and AREG, when compared to control Caco‐2 cells with uninhibited mTORC1 signalling (Figure 1d), we tested whether sEV preparations from these former cells might also stimulate Caco‐2 cell growth in an AREG‐dependent fashion. Indeed, Rab11a‐exosome‐enriched sEVs secreted from Torin1‐treated Caco‐2 cells produced mild growth‐promoting effects on Caco‐2 cells cultured in low serum and these effects were selectively inhibited following incubation with an anti‐AREG antibody (Figure 2f,g), suggesting that AREG associated with stress‐induced Rab11a‐exosomes from multiple CRC cell lines can promote growth.

Overall, we conclude that under stress conditions, CRC cells secrete increased levels of Rab11a‐exosomes, which can stimulate the growth of their neighbours through paracrine signalling involving exosome‐associated AREG.

### 3.3 Rab11a‐exosomes can induce resistance to cetuximab through an AREG‐dependent mechanism

EGFR‐targeted monoclonal antibodies, such as cetuximab, are used to treat KRAS‐wild type metastatic colorectal cancer, but eventually, resistance to this therapy emerges (Bhattacharya, 2023; Sforza et al., 2016). Since sEVs from CRC cells have previously been reported to induce cetuximab resistance (Yang et al., 2018; Zhang et al., 2017), we set out to test whether HCT116 sEVs, and in particular Rab11a‐exosomes, might mediate such effects.

Treatment of KRAS‐mutant HCT116 cells and KRAS‐wild type Caco‐2 cells with cetuximab under serum‐replete and ‐depleted conditions confirmed that while there was no effect on HCT116 cell growth (Figure S6a,b), Caco‐2 cell growth was sensitive to this drug under both conditions (Figure S6c). Furthermore, analysis of the phosphorylation state of both the EGFR and downstream ERK in these two cell lines under reduced serum conditions demonstrated that cetuximab selectively inhibited EGFR signalling in Caco‐2 cells (Figure S6d).

**FIGURE 3:**
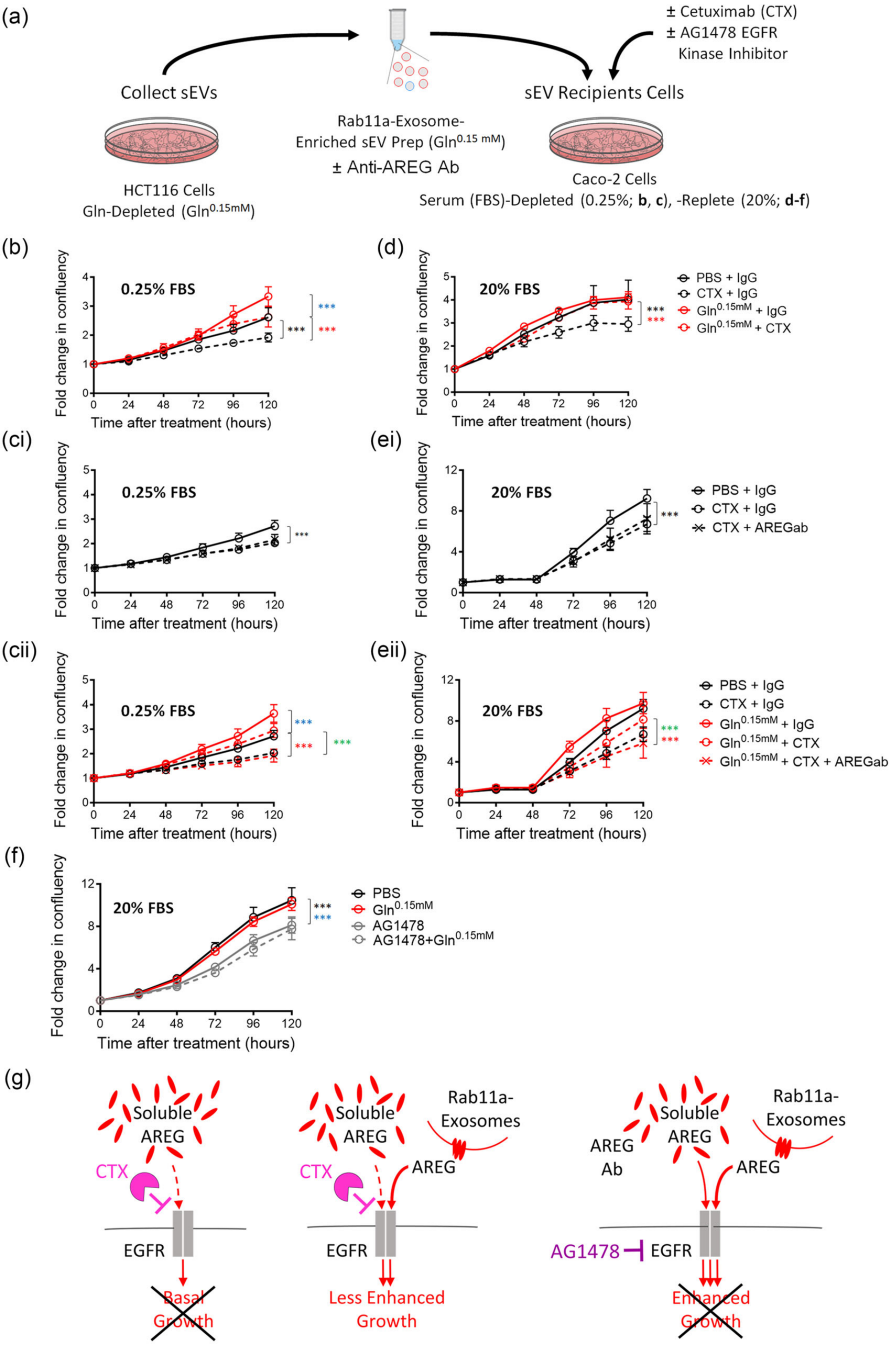
Rab11a‐exosome preparations induce AREG‐dependent cetuximab resistance in Caco‐2 cells. (a) Rab11a‐exosome‐enriched sEV preparations isolated from HCT116 cells were tested for their effects on cetuximab resistance in Caco‐2 cells, both in serum‐depleted (0.25% FBS) and serum‐replete (20% FBS) conditions, in the absence or presence of anti‐AREG antibodies and an EGFR kinase inhibitor. In b‐e, red and black lines on growth curves represent cells treated with Rab11a‐exosome‐enriched sEVs and PBS respectively, and dashed lines represent cetuximab‐treated cells. (b) HCT116 Rab11a‐exosome‐enriched sEV preparations suppress the growth inhibitory effect of cetuximab on Caco‐2 cells in serum‐depleted (0.25% FBS) conditions. (ci) Addition of anti‐AREG antibodies (AREG ab) to control Caco‐2 cells (PBS) treated with cetuximab has no further growth inhibitory effect on cetuximab‐treated cells (data points marked with crosses). (cii) In the same experiment, however, these antibodies completely suppress the growth‐promoting and cetuximab‐resistant effects of Rab11a‐exosome preparations (red lines). Control cell growth curves with and without cetuximab (black lines) are included in this graph for comparison. (d) HCT116 Rab11a‐exosome‐enriched sEV preparations suppress the growth inhibitory effect of cetuximab on Caco‐2 cells in serum‐replete (20% FBS) conditions, where in the absence of cetuximab, they have no additional growth‐promoting activity. (ei) Addition of anti‐AREG antibodies to control Caco‐2 cells has no further growth inhibitory effect on cetuximab‐treated cells. (eii) In the same experiment, however, these antibodies completely suppress the growth‐promoting and cetuximab‐resistant effects of Rab11a‐exosome preparations (red lines). Control cell growth curves with and without cetuximab are included in this graph for comparison (black lines). (f) The growth‐promoting effects of HCT116 Rab11a‐exosome preparations are completely suppressed by the EGFR kinase inhibitor AG1478. (g) Schematic models illustrating the effect of Rab11a‐exosomes, carrying the EGFR ligand AREG (middle), but not AREG in serum (left), in promoting cetuximab resistance in Caco‐2 CRC cells. Addition of either anti‐AREG antibodies (not shown) or the EGFR kinase inhibitor AG1478 (right) blocks the cetuximab resistance induced by Rab11a‐exosomes. Eight technical repeats were employed for each condition and each experiment was repeated three times. ***P < 0.001. In b–e, blue asterisks denote that sEV‐treated cell growth is significantly increased compared to PBS control. Red and green asterisks denote that sEV‐ and cetuximab‐treated cell growth is significantly increased compared to PBS control cells treated with cetuximab, and sEV‐/cetuximab‐treated cells after sEV pre‐incubation with anti‐AREG antibodies respectively. In f), black (control) and blue (sEV‐treated) asterisks denote significantly reduced cell growth after AG1478 treatment.

In cases where clones of cells carrying activating KRAS mutations emerge in tumours that are resistant to anti‐EGFR therapies, it is notable that most of the cells in the tumour do not carry this mutation (Misale et al., 2012). We hypothesised that sEVs, and in particular Rab11a‐exosomes, released from small numbers of cetuximab‐resistant CRC cells under cellular stress, might transfer resistance to cetuximab‐sensitive cells, permitting the survival and growth of these cells through paracrine signalling. We therefore undertook growth assays, incubating recipient Caco‐2 cells in serum‐replete and ‐depleted conditions with or without stress‐induced sEVs from HCT116 cells, and in the presence or absence of cetuximab (Figure 3a).

In low (0.25%) serum conditions, addition of stress‐induced sEVs from glutamine‐depleted HCT116 cells significantly stimulated the growth of Caco‐2 cells, relative to addition of PBS alone (Figure 3b). We have previously shown that these sEV preparations from HCT116 cells carry pg/ml concentrations of sEV‐associated AREG (Fan et al., 2020). Reproducing these growth‐promoting effects with recombinant AREG required ng/ml concentrations (Figure S7a,b), confirming our previous finding that Rab11a‐exosomes induce growth at much lower concentrations of AREG than soluble ligand.

Cetuximab reduced the growth of control (PBS) cells, suggesting that part of the growth observed in low serum conditions is stimulated by EGFR ligands. Cetuximab also reduced the growth induced by Rab11a‐exosomes, but only to levels observed in control cells that were not treated with cetuximab (Figure 3b). Growth was significantly enhanced compared to cells treated with PBS and cetuximab, indicating that stress‐induced sEVs induce cetuximab resistance. Importantly, although adding an anti‐AREG antibody had no additional growth‐inhibitory effect on cells treated with PBS and cetuximab (Figure 3ci), it further suppressed growth in the presence of HCT116 Rab11a‐exosomes and cetuximab to the same levels as cetuximab‐treated control cells (Figure 3cii). This suggests that the cetuximab resistance mediated by these vesicles is AREG‐dependent and therefore associated with Rab11a‐exosomes.

In high (20%) serum conditions, addition of HCT116 Rab11a‐exosome preparations had no significant effect on the growth of Caco‐2 cells relative to PBS addition, presumably because growth was already maximally stimulated by growth factors in 20% serum (Figure 3d). Cetuximab, however, had a completely different effect on cell growth in high serum in the presence and absence of Rab11a‐exosomes. While it strongly suppressed growth in control (PBS) Caco‐2 cells, it had no significant effect on growth in the presence of stress‐induced HCT116 sEVs (Figure 3d). As in low serum conditions, addition of an anti‐AREG antibody had no further growth‐inhibitory effect on control (PBS) Caco‐2 cells (Figure 3ei), but it reduced the growth of Caco‐2 cells in the presence of HCT116 sEVs to the levels observed in cetuximab‐treated control cells (Figure 3eii).

Taken together, these data indicate that unlike soluble AREG, HCT116 stress‐induced sEV preparations containing AREG on Rab11a‐exosomes confer cetuximab resistance to KRAS‐wild type Caco‐2 cells by competing with cetuximab at the EGFR (summarised in Figure 3g). Even in high serum, where Rab11a‐exosomes have no additional effect on growth, serum‐mediated growth is completely suppressed by cetuximab, while in the presence of Rab11a‐exosomes, growth, which appears to be induced by AREG‐dependent EGFR activation, is essentially unaffected.

There are, however, other interpretations. In particular, AREG on Rab11a‐exosomes could be acting via a receptor other than the EGFR, or the anti‐AREG antibody could indirectly block another signalling activity on Rab11a‐exosomes when it binds at the surface of these vesicles. We reasoned that in either of these scenarios, the HCT116 Rab11a‐exosome preparations would drive cell growth, even if the EGFR was blocked in a way that did not directly compete with AREG. To test this, we added the EGFR kinase inhibitor, AG1478 (Bishop et al., 2002), to Caco‐2 cells treated with PBS or HCT116 Rab11a‐exosomes. In this case, growth was similarly inhibited (Figure 3f, g), demonstrating that the effects of Rab11a‐exosomes on growth are being mediated through the EGFR, even though they are cetuximab‐resistant. The pan‐HER receptor kinase inhibitor BMS‐599626 (Wong et al., 2006) also completely blocked the growth‐promoting effects of HCT116 Rab11a‐exosome preparations (Figure S7c,d), consistent with this interpretation.

## 4 DISCUSSION

The emergence of cetuximab resistance is an inevitable outcome of cetuximab treatment of KRAS‐wild type tumours in metastatic CRC patients. It either involves the amplification of CRC cells carrying an activated KRAS mutation, which constitute a minority clone within a heterogeneous group of wild type KRAS cells (Misale et al., 2012), or the development of cetuximab resistance through microenvironmental changes (Woolston et al., 2019). Whatever the explanation, clonal heterogeneity and paracrine signalling appear to play a key role in this process (Chan & Buczaki, 2021). Indeed, Hobor et al. (2014) have reported an important function for EGFR ligands like AREG in mediating the communication between KRAS‐mutant and KRAS‐wild type cells in heterogeneous cetuximab‐resistant CRC tumours.

We have shown that this resistance can be mediated by AREG‐loaded, Rab11a‐exosomes produced by CRC cells under nutrient or hypoxic stresses, which are commonly experienced by growing tumours. We have previously demonstrated that AREG on these vesicles can enhance CRC growth at concentrations of approximately 1 pg/ml under conditions where soluble AREG requires concentrations three orders of magnitude higher (Fan et al., 2020). Here, we find that even under serum‐replete conditions, where addition of Rab11a‐exosomes from mutant KRAS HCT116 cells has no additional growth‐promoting effect on KRAS‐wild type Caco‐2 cells, these exosomes, but not soluble AREG, can compete with cetuximab for EGFR binding and activation. We suggest that the mechanism by which membrane‐bound AREG is presented to the EGFR, which may involve AREG clustering or association with other surface proteins, allows this ligand to circumvent the cetuximab blockade, which is normally mediated by high affinity monoclonal antibody binding. Delivery of AREG to target cells in Rab11a‐exosome or soluble forms may explain why high levels of AREG have been associated both with improved and poorer outcomes in response to cetuximab treatment (Hong et al., 2020; Randon & Pietrantonio, 2023; Williams et al., 2023).

**FIGURE 4:**
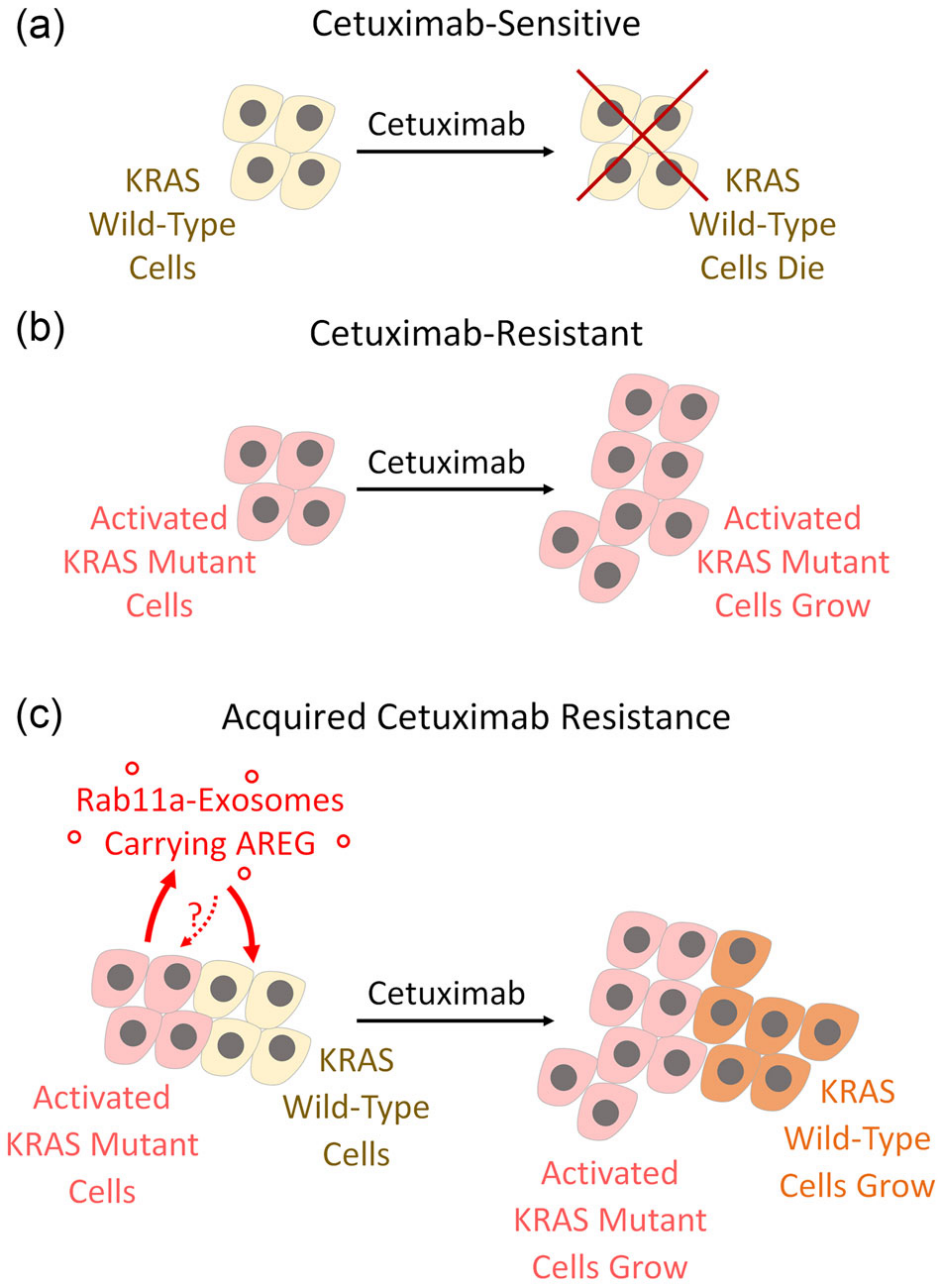
Schematic model highlighting the role of AREG on Rab11a‐exosomes in promoting cetuximab‐resistance in colorectal cancer. (a) The monoclonal antibody cetuximab (CTX) is typically effective in treating KRAS‐wild type colorectal cancer (CRC) cells (yellow cells). (b) Cetuximab is ineffective on other CRC cells that carry an active KRAS mutation (pink cells). These cells may produce Rab11a‐exosomes under nutrient and hypoxic stress, but they are presumably not required to maintain cetuximab resistance. (c) In heterogeneous CRC tumours, stress‐induced AREG‐loaded Rab11a‐exosomes from CTX‐resistant cells contribute to a mechanism leading to the development of drug resistance in previously CTX‐responsive cells, supporting clonal heterogeneity during tumour progression and further evolutionary change (orange cells).

Previous studies have suggested that CRC exosomes can promote cetuximab resistance in an apparently AREG‐independent fashion via the PTEN/Akt pathway, another target pathway of EGFR (Zhang et al., 2017), or via the transfer of the long non‐coding RNA uroepithelial carcinoma‐associated‐1 (UCA‐1) (Yang et al., 2018). Our data suggest an alternative mechanism that may explain how KRAS‐mutant cells could promote survival of KRAS‐wild type cells in a heterogeneous tumour (Figure 4). These findings suggest that treatments that either block Rab11a‐exosome biogenesis or suppress the active cargos on these exosomes might act synergistically with anti‐EGFR therapies to inhibit tumour growth and either slow or halt recurrence. In this regard, it is interesting to note that other forms of drug resistance have been attributed to exosome‐associated ligands, such a resistance to immune checkpoint inhibitors that re‐activate the patient's anti‐tumour response (Chen et al., 2018; Poggio et al., 2019). It will be interesting to explore whether stress‐induced Rab11a‐exosomes are involved in this resistance mechanism and whether selectively suppressing the production of these vesicles might provide a new therapeutic strategy.

## AUTHOR CONTRIBUTIONS

**John D. Mason**: Conceptualization; data curation; formal analysis; investigation; methodology; supervision; validation; visualization; writing—original draft; writing—review and editing. **Ewan Marks**: Data curation; formal analysis; investigation; methodology; validation; writing—review and editing. **Shih‐Jung Fan**: Conceptualization; data curation; formal analysis; methodology; resources; supervision; validation; visualization; writing—review and editing. **Kristie McCormick**: Methodology; resources; validation; writing—review and editing. **Clive Wilson**: Conceptualization; funding acquisition; project administration; supervision; validation; visualization; writing—original draft; writing—review and editing. **Adrian L. Harris**: Conceptualization; funding acquisition; project administration; supervision; validation; visualization; writing—review and editing. **Freddie C. Hamdy**: Conceptualization; funding acquisition; supervision; validation; writing—review and editing. **Chris Cunningham**: Conceptualization; funding acquisition; supervision; validation; writing—review and editing. **Deborah C. I. Goberdhan**: Conceptualization; data curation; formal analysis; funding acquisition; project administration; supervision; validation; visualization; writing—original draft; writing—review and editing.

## CONFLICTS OF INTEREST STATEMENT

The authors declare that they have no conflicts of interest in relation to this work.

## Supporting information

Supporting Information

## References

[jev212465-bib-0001] Ahmed, D. , Eide, P. W. , Eilertsen, I. A. , Danielsen, S. A. , Eknæs, M. , Hektoen, M. , Lind, G. E. , & Lothe, R. A. (2013). Epigenetic and genetic features of 24 colon cancer cell lines. Oncogenesis, 2, e71. 24042735 10.1038/oncsis.2013.35 PMC3816225

[jev212465-bib-0002] Allegra, C. J. , Rumble, R. B. , Hamilton, S. R. , Mangu, P. B. , Roach, N. , Hantel, A. , & Schilsky, R. L. (2016). Extended RAS gene mutation testing in metastatic colorectal carcinoma to predict response to anti‐epidermal growth factor receptor monoclonal antibody therapy: American Society of Clinical Oncology Provisional Clinical Opinion Update 2015. Journal of Clinical Oncology, 34, 179–185. 26438111 10.1200/JCO.2015.63.9674

[jev212465-bib-0043] Bando, H. , Ohtsu, A. , & Yoshino, T. (2023). Therapeutic landscape and future direction of metastatic colorectal cancer. Nature Reviews Gastroenterology & Hepatology, 20, 306–322. 36670267 10.1038/s41575-022-00736-1

[jev212465-bib-0003] Bhattacharya, S. (2023). An empirical review on the resistance mechanisms of epidermal growth factor receptor inhibitors and predictive molecular biomarkers in colorectal cancer. Critical Reviews in Oncology/Hematology, 183, 103916. 36717006 10.1016/j.critrevonc.2023.103916

[jev212465-bib-0004] Bishop, P. C. , Myers, T. , Robey, R. , Fry, D. W. , Liu, E. T. , Blagosklonny, M. V. , & Bates, S. E. (2002). Differential sensitivity of cancer cells to inhibitors of the epidermal growth factor receptor family. Oncogene, 21, 119–127. 11791182 10.1038/sj.onc.1205028

[jev212465-bib-0005] Cancer Research UK . (2023). https://www.cancerresearchuk.org/health‐professional/cancer‐statistics/statistics‐by‐cancer‐type/bowel‐cancer

[jev212465-bib-0006] Chan, D. K. H. , & Buczacki, S. J. A. (2021). Tumour heterogeneity and evolutionary dynamics in colorectal cancer. Oncogenesis, 10, 53. 34272358 10.1038/s41389-021-00342-x PMC8285471

[jev212465-bib-0007] Chen, G. , Huang, A. C. , Zhang, W. , Zhang, G. , Wu, M. , Xu, W. , Yu, Z. , Yang, J. , Wang, B. , Sun, H. , Xia, H. , Man, Q. , Zhong, W. , Antelo, L. F. , Wu, B. , Xiong, X. , Liu, X. , Guan, L. , Li, T. , … Guo, W. (2018). Exosomal PD‐L1 contributes to immunosuppression and is associated with anti‐PD‐1 response. Nature, 560, 382–386. 30089911 10.1038/s41586-018-0392-8 PMC6095740

[jev212465-bib-0008] Cheng, L. , & Hill, A. F. (2022). Therapeutically harnessing extracellular vesicles. Nature Reviews Drug Discovery, 21, 379–399. 35236964 10.1038/s41573-022-00410-w

[jev212465-bib-0009] Corrigan, L. , Redhai, S. , Leiblich, A. , Fan, S. J. , Perera, S. M. , Patel, R. , Gandy, C. , Wainwright, S. M. , Morris, J. F. , Hamdy, F. , Goberdhan, D. C. , & Wilson, C. (2014). BMP‐regulated exosomes from *Drosophila* male reproductive glands reprogram female behavior. Journal of Cell Biology, 206, 671–688. 25154396 10.1083/jcb.201401072 PMC4151142

[jev212465-bib-0011] Dixson, A. C. , Dawson, T. R. , di Vizio, D. , & Weaver, A. M. (2023). Context‐specific regulation of extracellular vesicle biogenesis and cargo selection. Nature Reviews Molecular Cell Biology, 24, 454–476. 36765164 10.1038/s41580-023-00576-0 PMC10330318

[jev212465-bib-0012] Fan, S. J. , Kroeger, B. , Marie, P. P. , Bridges, E. M. , Mason, J. D. , McCormick, K. , Zois, C. E. , Sheldon, H. , Khalid Alham, N. , Johnson, E. , Ellis, M. , Stefana, M. I. , Mendes, C. C. , Wainwright, S. M. , Cunningham, C. , Hamdy, F. C. , Harris, A. L. , Wilson, C. , & Goberdhan, D. C. I. (2020). Glutamine deprivation alters the origin and function of cancer cell exosomes. Embo Journal, 39, e1039009. 10.15252/embj.2019103009 PMC7429491 32720716

[jev212465-bib-0013] Flecchia, C. , Zaanan, A. , Lahlou, W. , Basile, D. , Broudin, C. , Gallois, C. , Pilla, L. , Karoui, M. , Manceau, G. , & Taieb, J. (2022). MSI colorectal cancer, all you need to know. Clinics and Research in Hepatology and Gastroenterology, 46, 101983. 35732266 10.1016/j.clinre.2022.101983

[jev212465-bib-0014] Fornasier, G. , Francescon, S. , & Baldo, P. (2018). An update of efficacy and safety of cetuximab in metastatic colorectal cancer: A narrative review. Advances in Therapy, 35, 1497–1509. 30218345 10.1007/s12325-018-0791-0

[jev212465-bib-0015] Goberdhan, D. C. , Wilson, C. , & Harris, A. L. (2016). Amino Acid Sensing by mTORC1: Intracellular Transporters Mark the Spot. Cell Metabolism, 23, 580–589. 27076075 10.1016/j.cmet.2016.03.013 PMC5067300

[jev212465-bib-0016] Guinney, J. , Dienstmann, R. , Wang, X. , de Reyniès, A. , Schlicker, A. , Soneson, C. , Marisa, L. , Roepman, P. , Nyamundanda, G. , Angelino, P. , Bot, B. M. , Morris, J. S. , Simon, I. M. , Gerster, S. , Fessler, E. , De Sousa E Melo, F. , Missiaglia, E. , Ramay, H. , Barras, D. , … Tejpar, S. (2015). The consensus molecular subtypes of colorectal cancer. Nature Medicine, 21, 1350–1356. 10.1038/nm.3967 PMC4636487 26457759

[jev212465-bib-0017] Hobor, S. , Van Emburgh, B. O. , Crowley, E. , Misale, S. , Di Nicolantonio, F. , & Bardelli, A. (2014). TGFα and amphiregulin paracrine network promotes resistance to EGFR blockade in colorectal cancer cells. Clinical Cancer Research, 20, 6429–6438. 24916700 10.1158/1078-0432.CCR-14-0774

[jev212465-bib-0018] Hong, C. S. , Sun, E. G. , Choi, J. N. , Kim, D. H. , Kim, J. H. , Ryu, K. H. , Shim, H. J. , Hwang, J. E. , Bae, W. K. , Kim, H. R. , Kim, K. K. , Jung, C. , Chung, I. J. , & Cho, S. H. (2020). Fibroblast growth factor receptor 4 increases epidermal growth factor receptor (EGFR) signaling by inducing amphiregulin expression and attenuates response to EGFR inhibitors in colon cancer. Cancer Science, 111, 3268–3278. 32533590 10.1111/cas.14526 PMC7469799

[jev212465-bib-0019] Jeppesen, D. K. , Fenix, A. M. , Franklin, J. L. , Higginbotham, J. N. , Zhang, Q. , Zimmerman, L. J. , Liebler, D. C. , Ping, J. , Liu, Q. , Evans, R. , Fissell, W. H. , Patton, J. G. , Rome, L. H. , Burnette, D. T. , & Coffey, R. J. (2019). Reassessment of exosome composition. Cell, 177, 428–445.e18. 30951670 10.1016/j.cell.2019.02.029 PMC6664447

[jev212465-bib-0020] Kucharzewska, P. , Christianson, H. C. , Welch, J. E. , Svensson, K. J. , Fredlund, E. , Ringnér, M. , Mörgelin, M. , Bourseau‐Guilmain, E. , Bengzon, J. , & Belting, M. (2013). Exosomes reflect the hypoxic status of glioma cells and mediate hypoxia‐dependent activation of vascular cells during tumor development. PNAS, 110, 7312–7317. 23589885 10.1073/pnas.1220998110 PMC3645587

[jev212465-bib-0021] Lucotti, S. , Kenific, C. M. , Zhang, H. , & Lyden, D. (2022). Extracellular vesicles and particles impact the systemic landscape of cancer. Embo Journal, 41, e109288. 36052513 10.15252/embj.2021109288 PMC9475536

[jev212465-bib-0023] Marie, P. P. , Fan, S. J. , Mason, J. D. , Wells, A. , Mendes, C. C. , Wainwright, S. M. , Scott, S. , Fischer R Harris, A. L. , Wilson, C. , & Goberdhan, D. C. I. (2023). Accessory ESCRT‐III proteins are conserved and selective regulators of Rab11a‐exosome formation. Journal of Extracellular Vesicles, 12, e12311. 36872252 10.1002/jev2.12311 PMC9986085

[jev212465-bib-0024] Misale, S. , Yaeger, R. , Hobor, S. , Scala, E. , Janakiraman, M. , Liska, D. , Valtorta, E. , Schiavo, R. , Buscarino, M. , Siravegna, G. , Bencardino, K. , Cercek, A. , Chen, C. T. , Veronese, S. , Zanon, C. , Sartore‐Bianchi, A. , Gambacorta, M. , Gallicchio, M. , Vakiani, E. , … Bardelli, A. (2012). Emergence of KRAS mutations and acquired resistance to anti‐EGFR therapy in colorectal cancer. Nature, 486, 532–536. 22722830 10.1038/nature11156 PMC3927413

[jev212465-bib-0025] Poggio, M. , Hu, T. , Pai, C. C. , Chu, B. , Belair, C. D. , Chang, A. , Montabana, E. , Lang, U. E. , Fu, Q. , Fong, L. , & Blelloch, R. (2019). Suppression of Exosomal PD‐L1 Induces Systemic Anti‐tumor Immunity and Memory. Cell, 177, 414–427.e13. 30951669 10.1016/j.cell.2019.02.016 PMC6499401

[jev212465-bib-0026] Raimondo, S. , Saieva, L. , Vicario, E. , Pucci, M. , Toscani, D. , Manno, M. , Raccosta, S. , Giuliani, N. , & Alessandro, R. (2019). Multiple myeloma‐derived exosomes are enriched of amphiregulin (AREG) and activate the epidermal growth factor pathway in the bone microenvironment leading to osteoclastogenesis. Journal of Hematology & Oncology, 12, 2. 30621731 10.1186/s13045-018-0689-y PMC6325886

[jev212465-bib-0027] Randon, G. , & Pietrantonio, F. (2023). Towards multiomics‐based dissection of anti‐EGFR sensitivity in colorectal cancer. Clinical Cancer Research, 29, 4021–4023. 37594733 10.1158/1078-0432.CCR-23-1954 PMC10570674

[jev212465-bib-0029] Sforza, V. , Martinelli, E. , Ciardiello, F. , Gambardella, V. , Napolitano, S. , Martini, G. , Della Corte, C. , Cardone, C. , Ferrara, M. L. , Reginelli, A. , Liguori, G. , Belli, G. , & Troiani, T. (2016). Mechanisms of resistance to anti‐epidermal growth factor receptor inhibitors in metastatic colorectal cancer. World Journal of Gastroenterology, 22, 6345–6361. 27605871 10.3748/wjg.v22.i28.6345 PMC4968117

[jev212465-bib-0030] Sveen, A. , Bruun, J. , Eide, P. W. , Eilertsen, I. A. , Ramirez, L. , Murumägi, A. , Arjama, M. , Danielsen, S. A. , Kryeziu, K. , Elez, E. , Tabernero, J. , Guinney, J. , Palmer, H. G. , Nesbakken, A. , Kallioniemi, O. , Dienstmann, R. , & Lothe, R. A. (2018). Colorectal cancer consensus molecular subtypes translated to preclinical models uncover potentially targetable cancer cell dependencies. Clinical Cancer Research, 24, 794–806. 29242316 10.1158/1078-0432.CCR-17-1234

[jev212465-bib-0031] van Niel, G. , Carter, D. R. F. , Clayton, A. , Lambert, D. W. , Raposo, G. , & Vader, P. (2022). Challenges and directions in studying cell‐cell communication by extracellular vesicles. Nature Reviews Molecular Cell Biology, 23, 369–382. 35260831 10.1038/s41580-022-00460-3

[jev212465-bib-0032] Vietri, M. , Radulovic, M. , & Stenmark, H. (2019). The many functions of ESCRTs. Nature Reviews Molecular Cell Biology, 21, 25–42. 31705132 10.1038/s41580-019-0177-4

[jev212465-bib-0033] Wang, J. , Mouradov, D. , Wang, X. , Jorissen, R. N. , Chambers, M. C. , Zimmerman, L. J. , Vasaikar, S. , Love, C. G. , Li, S. , Lowes, K. , Leuchowius, K. J. , Jousset, H. , Weinstock, J. , Yau, C. , Mariadason, J. , Shi, Z. , Ban, Y. , Chen, X. , Coffey, R. J. C. , … Sieber, O. M. (2017). Colorectal cancer cell line proteomes are representative of primary tumors and predict drug sensitivity. Gastroenterology, 153, 1082–1095. 28625833 10.1053/j.gastro.2017.06.008 PMC5623120

[jev212465-bib-0034] Welsh, J. A. , Goberdhan, D. C. I. , O'Driscoll, L. , Buzas, E. I. , Blenkiron, C. , Bussolati, B. , Cai, H. , Di Vizio, D. , Driedonks, T. A. P. , Erdbrügger, U. , Falcon‐Perez, J. M. , Fu, Q. L. , Hill, A. F. , Lenassi, M. , Lim, S. K. , Mahoney, M. G. , Mohanty, S. , Möller, A. , … Witwer, K. W. (2024). Minimal information for studies of extracellular vesicles (MISEV2023): From basic to advanced approaches. Journal of Extracellular Vesicles, e12404. 38326288 10.1002/jev2.12404 PMC10850029

[jev212465-bib-0035] Williams, C. J. M. , Elliott, F. , Sapanara, N. , Aghaei, F. , Zhang, L. , Muranyi, A. , Yan, D. , Bai, I. , Zhao, Z. , Shires, M. , Wood, H. M. , Richman, S. D. , Hemmings, G. , Hale, M. , Bottomley, D. , Galvin, L. , Cartlidge, C. , Dance, S. , Bacon, C. M. , … Quirke, P. (2023). Associations between AI‐Assisted Tumor Amphiregulin and Epiregulin IHC and Outcomes from Anti‐EGFR Therapy in the Routine Management of Metastatic Colorectal Cancer. Clinical Cancer Research, 29, 4153–4165. 37363997 10.1158/1078-0432.CCR-23-0859 PMC10570673

[jev212465-bib-0036] Wilson, C. , Leiblich, A. , Goberdhan, D. C. I. , & Hamdy, F. C. (2017). The *Drosophila* accessory gland as a model for prostate cancer and other pathologies. Current Topics in Developmental Biology, 121, 339–375. 28057306 10.1016/bs.ctdb.2016.06.001 PMC5224695

[jev212465-bib-0037] Wong, T. W. , Lee, F. Y. , Yu, C. , Luo, F. R. , Oppenheimer, S. , Zhang, H. , Smykla, R. A. , Mastalerz, H. , Fink, B. E. , Hunt, J. T. , Gavai, A. V. , & Vite, G. D. (2006). Preclinical antitumor activity of BMS‐599626, a pan‐HER kinase inhibitor that inhibits HER1/HER2 homodimer and heterodimer signaling. Clinical Cancer Research, 12, 6186–6193. 17062696 10.1158/1078-0432.CCR-06-0642

[jev212465-bib-0038] Woolston, A. , Khan, K. , Spain, G. , Barber, L. J. , Griffiths, B. , Gonzalez‐Exposito, R. , Hornsteiner, L. , Punta, M. , Patil, Y. , Newey, A. , Mansukhani, S. , Davies, M. N. , Furness, A. , Sclafani, F. , Peckitt, C. , Jiménez, M. , Kouvelakis, K. , Ranftl, R. , Begum, R. , … Gerlinger, M. (2019). Genomic and transcriptomic determinants of therapy resistance and immune landscape evolution during anti‐EGFR treatment in colorectal cancer. Cancer Cell, 36, 35–50. 31287991 10.1016/j.ccell.2019.05.013 PMC6617392

[jev212465-bib-0039] Yang, Y. N. , Zhang, R. , Du, J. W. , Yuan, H. H. , Li, Y. J. , Wei, X. L. , Du, X. X. , Jiang, S. L. , & Han, Y. (2018). Predictive role of UCA1‐containing exosomes in cetuximab‐resistant colorectal cancer. Cancer Cell International, 18, 164. 30377411 10.1186/s12935-018-0660-6 PMC6196422

[jev212465-bib-0040] Yates, A. G. , Pink, R. C. , Erdbrügger, U. , Siljander, P. R. , Dellar, E. R. , Pantazi, P. , Akbar, N. , Cooke, W. R. , Vatish, M. , Dias‐Neto, E. , Anthony, D. C. , & Couch, Y. (2022). In sickness and in health: The functional role of extracellular vesicles in physiology and pathology *in vivo* . Part I: Health and Normal Physiology Journal of Extracellular Vesicles, 11, e12151. 35041249 10.1002/jev2.12151 PMC8765331

[jev212465-bib-0041] Zhang, S. , Zhang, Y. , Qu, J. , Che, X. , Fan, Y. , Hou, K. , Guo, T. , Deng, G. , Song, N. , Li, C. , Wan, X. , Qu, X. , & Liu, Y. (2017). Exosomes promote cetuximab resistance via the PTEN/Akt pathway in colon cancer cells. Brazilian Journal of Medical and Biological Research, 51, e6472. 29160412 10.1590/1414-431X20176472 PMC5685060

