# Correction to “Stress‐Induced Rab11a‐Exosomes Induce Amphiregulin‐Mediated Cetuximab Resistance in Colorectal Cancer”

**DOI:** 10.1002/jev2.70081

**Published:** 2025-05-02

J.D. Mason, E. Marks, S.J. Fan, K. McCormick, C. Wilson, A.L. Harris, F.C. Hamdy, C. Cunningham, and D.C.I. Goberdhan, “Stress‐Induced Rab11a‐Exosomes Induce Amphiregulin‐Mediated Cetuximab Resistance in Colorectal Cancer,” *Journal of Extracellular Vesicles* 13, no. 6 (2024): e12465, https://doi.org/10.1002/jev2.12465.

In the original article, the data in Figure 1b, which had previously been published in Fan et al. 2020 *EMBO J* 39(16), e103009, was inadvertently included, in addition to citing this article. This panel has now been removed. Published below is the correct version of Figure 1 with its modified legend and a slightly modified version of the first results section text, in which this figure and the associated Figure S2 are cross‐referenced.

**FIGURE 1:**
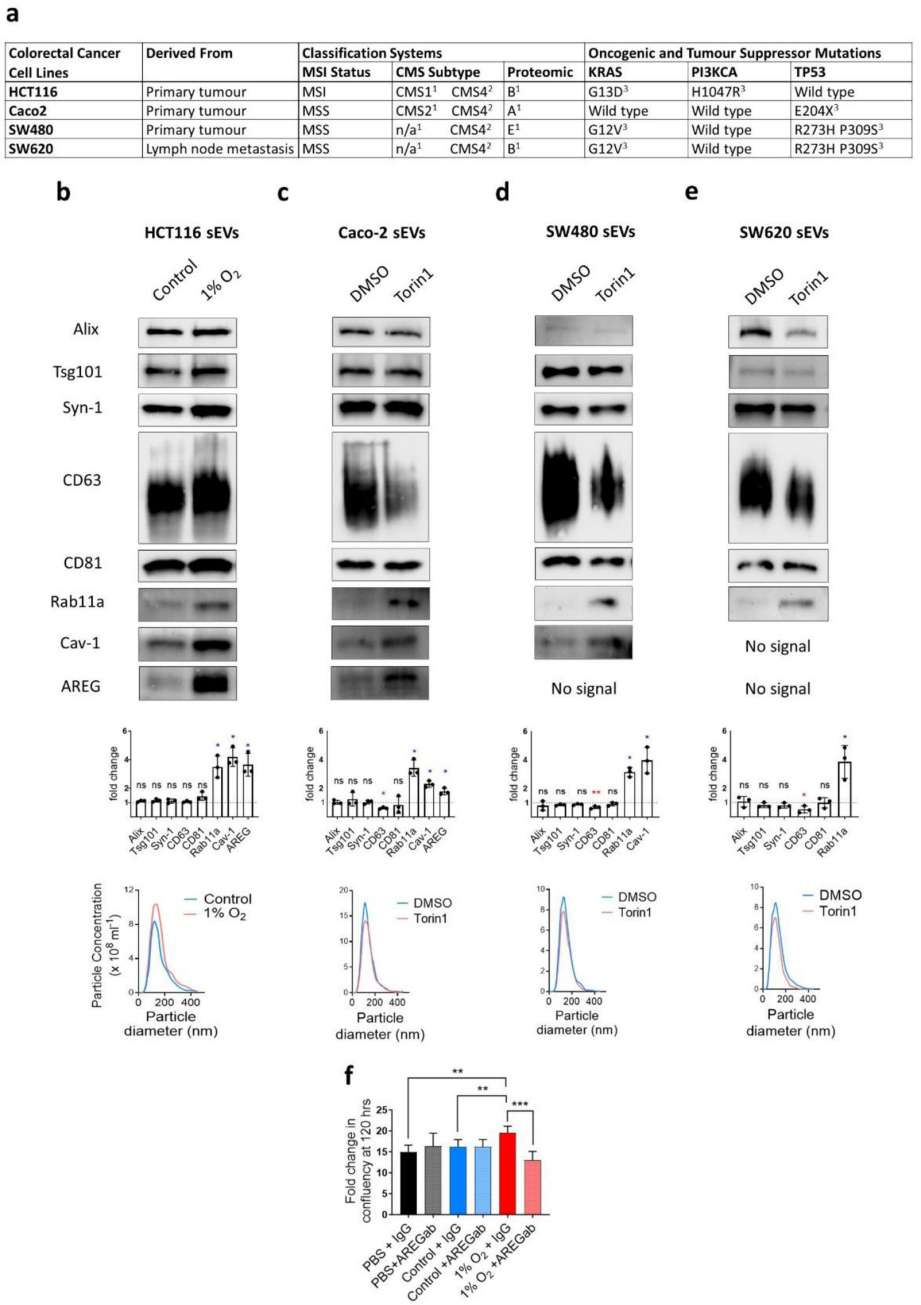
Inhibition of mTORC1 induces secretion of Rab11a‐exosomes in multiple CRC cell lines. (a) Classification of the four CRC cell lines investigated based on molecular pathways and mutational status in critical oncogenes and tumour suppressor genes. (b) Western blot analysis of sEV proteins isolated from HCT116 cells cultured in hypoxic (1% O_2_) versus normoxic conditions reveals a consistent increase in markers of Rab11a‐exosomes (Rab11a and AREG) and other stress‐induced sEVs (Cav‐1; see histogram, where changes in protein levels, measured by densitometry and normalised to protein levels in secreting cell lysates, are plotted for three independent experiments; mean ± SD). (c–e) Western blots of sEV proteins from Caco‐2 (c), SW480 (d) and SW620 (e) cells treated with the ATP‐competitive mTORC1 inhibitor, Torin1 (100 nM for SW480 cells and 150 nM for Caco‐2 and SW620 cells), versus treatment with vehicle alone (DMSO) also reveals a switch to secretion of increased Rab11a and AREG, in addition to a reduction in CD63. NTA reveals that all sEV preparations have similar size distribution, but particle numbers were generally slightly reduced, ie. SW480: 41.58 ± 0.73 × 10^8^ particles/mL (Torin1) versus 42.58 ± 1.74 × 10^8^ (control) particles/mL: SW620: 13.71 ± 0.47 × 10^8^ particles/mL (Torin1) versus 15.20 ± 0.37 × 10^8^ (control) particles/mL: Caco2: 14.37 ± 0.98 × 10^8^ particles/mL (Torin1) versus 18.11 ± 1.27 × 10^8^ (control) particles/mL. (f) Graph showing levels of HCT116 growth after 120 h, measured as fold change in confluency, following addition of PBS, control HCT116 sEVs, or hypoxia‐induced HCT116 sEVs in the presence or absence of pre‐incubation with neutralising anti‐AREG antibodies. Note that only sEVs isolated under hypoxic conditions enhance growth and this is AREG‐dependent. *P < 0.05. Red and blue asterisks denote reduction and increase in protein levels respectively in Rab11a‐exosome‐enriched sEVs. MSI, microsatellite instability; MSS, microsatellite stability; CMS, consensus molecular subtype; n/a, not applicable. KRAS activating mutations: G13D or G12V. PI3K activating mutations: E545K D549N or H1047R. TP53 inactivating mutations: R273H P309S, or S241F, or E204X. Superscripts 1,2,3 refer to ^1^Ahmed et al. (2013); ^2^Sveen et al. (2018); ^3^Wang et al. (2017).

In the original article, the data in Figure S2b, which was associated with Figure 1b and had previously been published in Fan et al. 2020 *EMBO J* 39(16), e103009, was inadvertently included. This has now been removed, and the corrected version of this figure is included in the new Supplementary Information file.

In the original article, the wrong tubulin blot was mistakenly included in Figure S3g. This has now been corrected and included in the new Supplementary Information file.

These mistakes do not affect the significance of the findings or the conclusions in this article. We apologise for these errors.

In HCT116 cells, downregulation of the mTORC1 signalling pathway in response to glutamine depletion leads to a switch to increased secretion of Rab11a‐exosomes carrying membrane‐bound AREG and Rab11a. It does not, however, affect the levels of other exosome and EV proteins in sEV preparations, except for late endosomal marker CD63, which is reduced under stress conditions (Fan et al., 2020; Marie et al., 2023). Experiments using immuno‐affinity separation of sEVs and selective inhibition of Rab11a‐exosome secretion suggest that unlike AREG, scaffolding protein Cav‐1, which is also elevated in Rab11a‐exosome‐enriched sEV preparations, is associated with alternative stress‐induced vesicles that co‐separate with Rab11a‐exosomes (Fan et al., 2020; Marie et al., 2023). The decrease in sEV‐associated CD63 is partially caused by a stress‐induced reduction in trafficking and exosome secretion through the late endosomal pathway (Fan et al., 2020).

Hypoxia, a common microenvironmental stress in fast‐growing tumours, previously shown to alter sEV cargos in glioblastoma cells (Kurcharzewska et al., 2013), also inhibited mTORC1 in HCT116 cells, as determined by reduced phospho‐S6 and phosphorylated forms of 4E‐BP1 in western blots of cell lysates (Figure S2b). While only the exosome and sEV proteins CD81 (decreased) and AREG (increased) were significantly changed in cell lysates (Figure S2b), hypoxia induced a significant increase in Rab11a, Cav‐1 and AREG in sEV preparations, consistent with elevated Rab11a‐exosome secretion (Figure 1b). In contrast to glutamine depletion, the levels of sEV‐associated CD63 were not reduced under hypoxia, suggesting that secretion of late endosomal exosomes was not reduced by this treatment. Analysis of SEC‐separated sEVs secreted from HCT116 cells under normal conditions by transmission electron microscopy (TEM) revealed the standard cup‐like morphology typically associated with sEV preparations (Figure S2f). Hypoxia‐induced vesicles preferentially promoted growth of HCT116 cells under serum‐depleted conditions, an activity that could be blocked by adding neutralising anti‐AREG antibodies to the sEV preparations (Figure 1f). A similar inhibitory effect was previously observed on Rab11a‐exosome‐enriched sEV preparations from glutamine‐depleted HCT116 cells (Fan et al., 2020) and suggests that the enhanced growth‐promoting effects are mediated by membrane‐associated AREG loaded on to hypoxia‐induced Rab11a‐exosomes.

For Caco‐2, SW480 and SW620 CRC cell lines, glutamine depletion did not affect mTORC1 activity in a dose‐response experiment, as determined by the phosphorylation state of downstream target 4E‐BP1, and only variably reduced S6 phosphorylation (Figure S3a–d). Each of these cell lines was, however, sensitive to the mTORC1 inhibitor Torin1 in the dose range of between 100–150 nM, which affected both S6 and 4E‐BP1 (Figure S3e–g). Under these conditions, Torin1 treatment induced an increase in sEV‐associated Rab11a and Cav‐1 (although not for SW620 cells, which lack Cav‐1), and a decrease in the tetraspanin CD63 (Figure 1c‐e), but without a change in levels of these proteins in cell lysates (except for reduced CD63 levels in Caco‐2 cells; Figure S2c‐e). Only sEVs from Caco‐2 cells contained detectable levels of AREG, but as with HCT116 cells, these levels were strongly increased following mTORC1 inhibition. In Caco‐2 cells, however, cellular levels of AREG were not affected by this treatment. Other EV markers, such as Alix, Tsg101, Syn‐1 and CD81, were unchanged in sEV preparations from all three cell types (Figure 1c‐e) and also in the secreting cells (Figure S2c‐e). For all three cell lines, Torin1 treatment did not significantly alter the size of vesicles in sEV preparations: Caco‐2, 139 ± 58 nm diameter (control) versus 143 ± 57 nm (Torin1); SW480, 149 ± 55 nm (control) versus 150 ± 49 nm (Torin1); SW620, 111 ± 54 nm (control) versus 109 ± 54 (Torin1). sEV secretion, however, was reduced by up to 20% for each of these cell lines, as determined by NTA (Figure 1c‐e). EVs from each cell line produced cup‐like particles typical of sEV morphology in TEM studies (Figure S2g‐i).

## Supporting information



Supporting Information

