# Supplementary material for: Stress‐induced Rab11a‐exosomes induce amphiregulin‐mediated cetuximab resistance in colorectal cancer
Source: J Extracell Vesicles. 2024 Jun 18;13(6):e12465. doi: 10.1002/jev2.12465 (PMC11184284; doi:10.1002/jev2.12465)
Supplement: Supplementary file 1 — Supporting Information [file JEV2-13-e12465-s001.docx]

**Supplementary Figures**


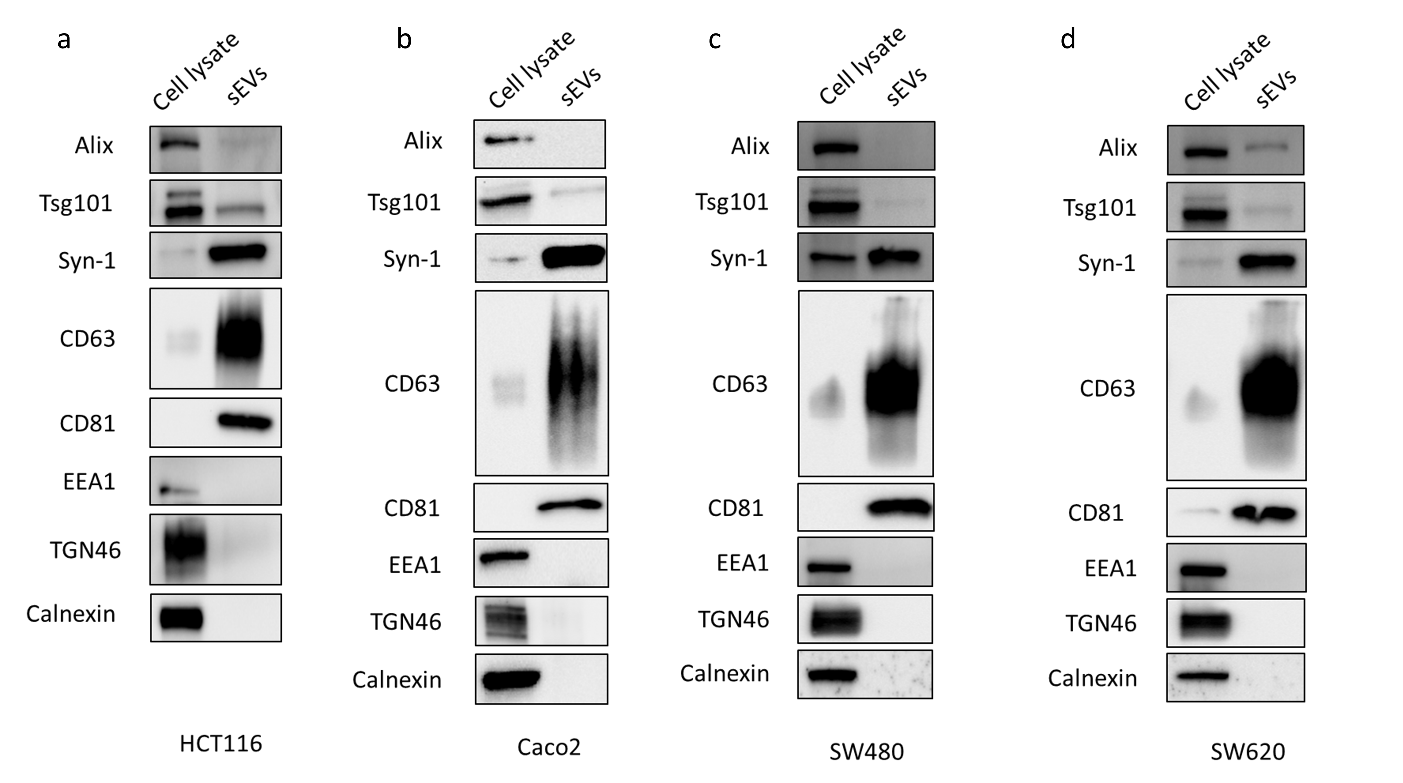


**Fig. S1. sEV preparations from CRC cell lines do not contain early endosomal, Golgi or ER membrane-associated markers.** (a-d) Western blots of sEV, early endosomal (EEA1), Golgi (TGN46) and ER (Calnexin) markers for cell lysates and sEVs from HCT116 (a), Caco2 (b), SW480 (c) and SW620 (d) cells.


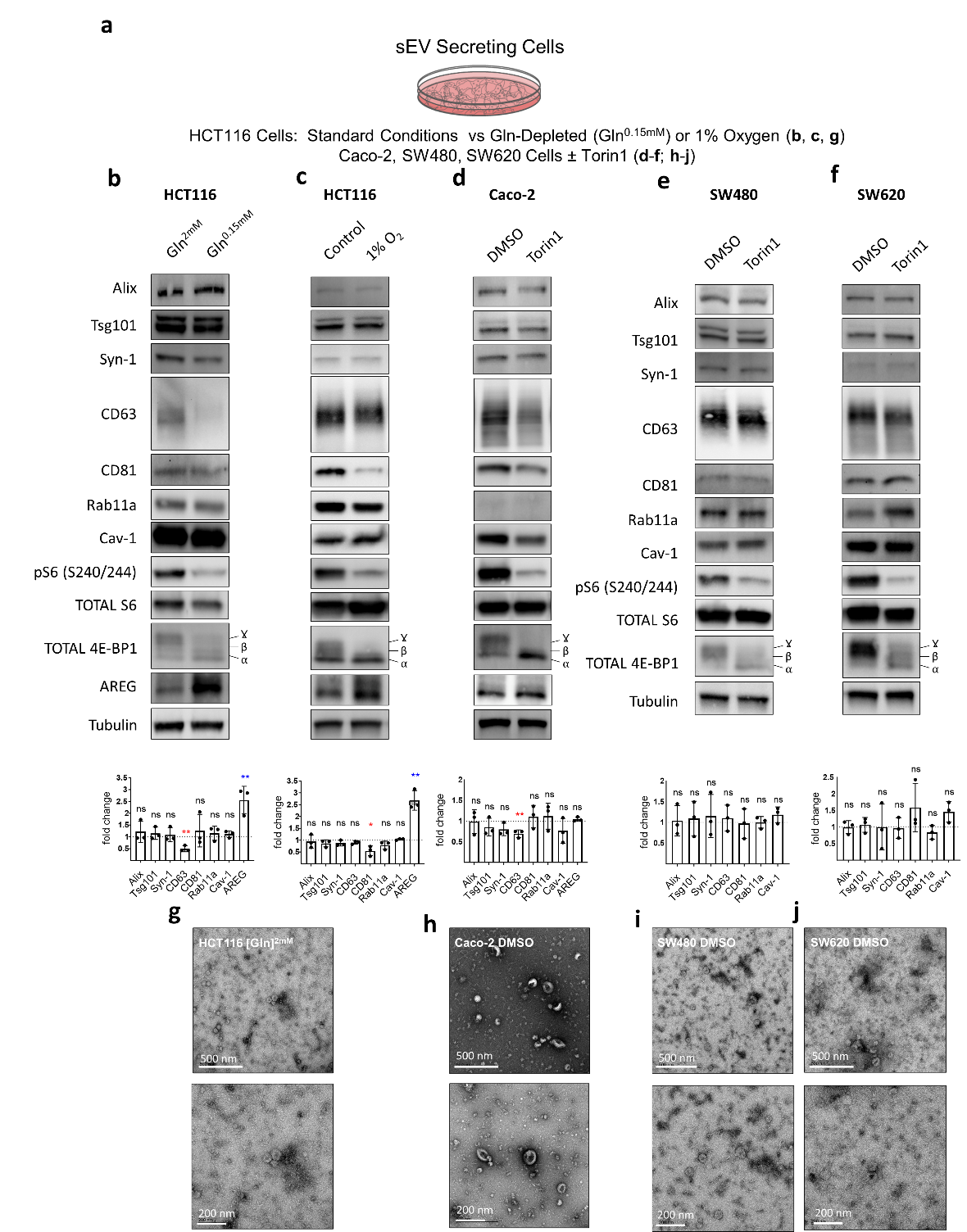


**Fig. S2. mTORC1 inhibition in CRC cell lines generally does not affect cellular expression levels of Rab11a and other sEV markers**. (a) Schematic of manipulations and drug treatments applied to cells and assessed by western analysis in (b-f) below. (b-f) Western blots of CRC cell lysates from cells incubated in mTORC1-inhibitory conditions that induce Rab11a-exosome secretion versus non-inhibitory conditions. The Rab11a-exosome-inducing conditions include glutamine depletion (HCT116 cells, 0.15 mM versus 2 mM; b), hypoxia (HCT116 cells, 1% hypoxia; c), and Torin1 treatment for Caco-2 (150 nM Torin1; d), SW480 (100 nM Torin1; e) and SW620 (150 nM Torin1; f) cells. Note reduced levels of phospho-S6 (P-S6) and phosphorylated forms of 4E-BP1 (α, β, and γ) under inhibitory conditions, as well as similar levels of many sEV markers (Alix, Tsg101, Syn-1, CD81, Rab11a, Cav-1) following most treatments, as shown in histograms (levels normalised to Tubulin; n = 3). CD63 expression is reduced when mTORC1 is inhibited in glutamine-depleted, but not hypoxic, HCT116 cells and in Caco-2 cells. (g-j) Transmission electron microscopy images of sEV preparations confirm that vesicles isolated from these different cells are less than 200 nm in diameter and reveal a classic cup-like morphology of vesicles prepared using this procedure. *P<0.05. Red and blue asterisks denote reduction and increase in protein levels respectively in mTORC1-inhibited cells.


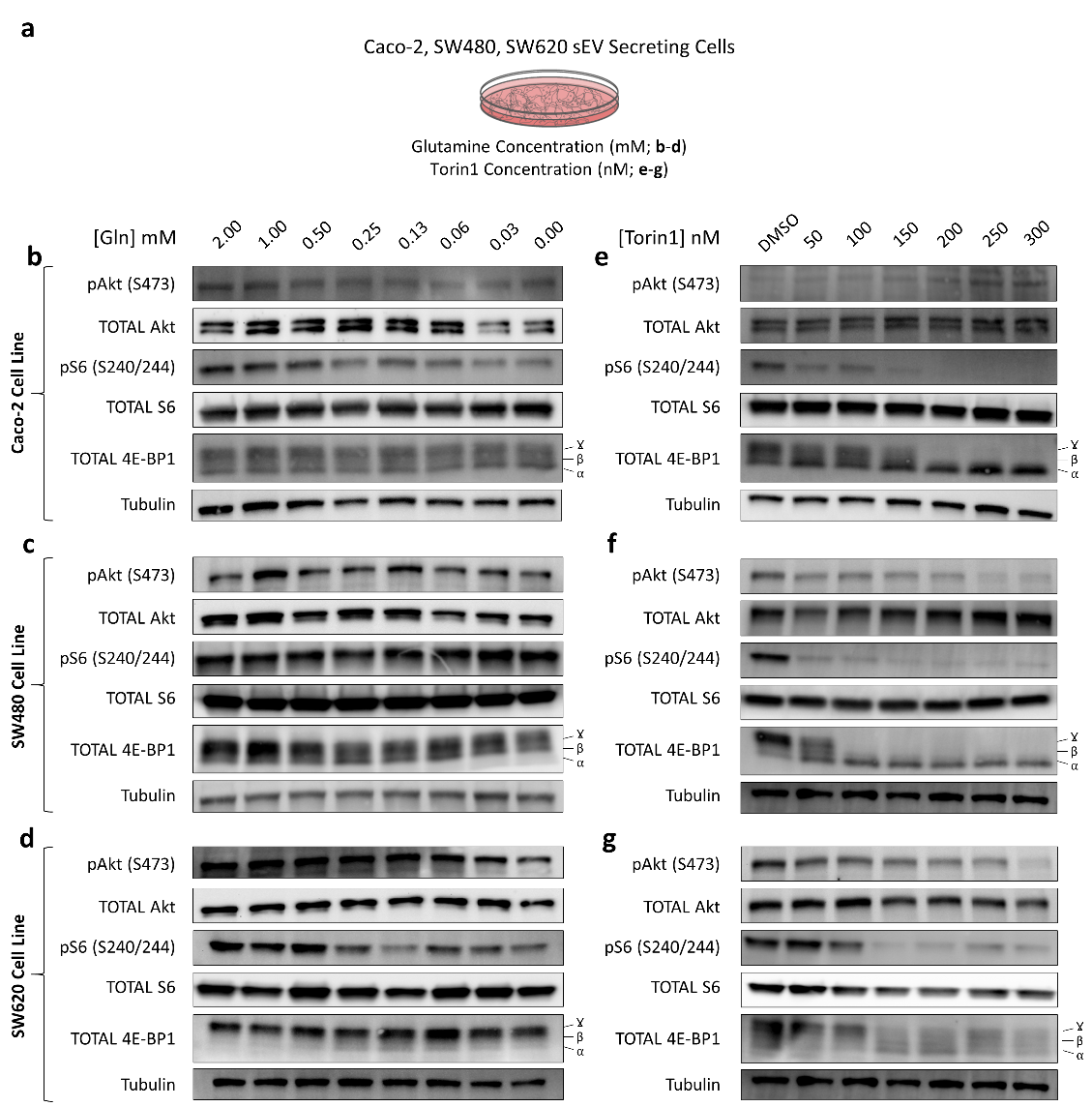


**Fig. S3. Torin1, but not glutamine depletion, inhibits mTORC1 activity in Caco-2, SW480 and SW620 cells.** (a) Experimental design relevant to the data below. (b-d) Western blots of cell lysates from Caco-2 (b), SW480 (c) and SW620 (d) cells cultured in different concentrations of glutamine reveal that the activity of Akt, and mTORC1 downstream targets, S6 and 4E-BP1, as measured by their phosphorylation, is largely unaffected by glutamine depletion. Phosphorylated forms of 4E-BP1 run slower in polyacrylamide gels, producing α, β, and γ forms. (e-g) Western blots of cell lysates from Caco-2 (e), SW480 (f) and SW620 (g) cells cultured in different concentrations of mTOR inhibitor Torin1, which inhibits mTOR-containing mTORC2 (a regulator of Akt) as well as mTORC1. These reveal that the activity of Akt, and mTORC1 downstream targets, S6 and 4E-BP1, as measured by their phosphorylation, is blocked by Torin1 in a dose-dependent manner.

**
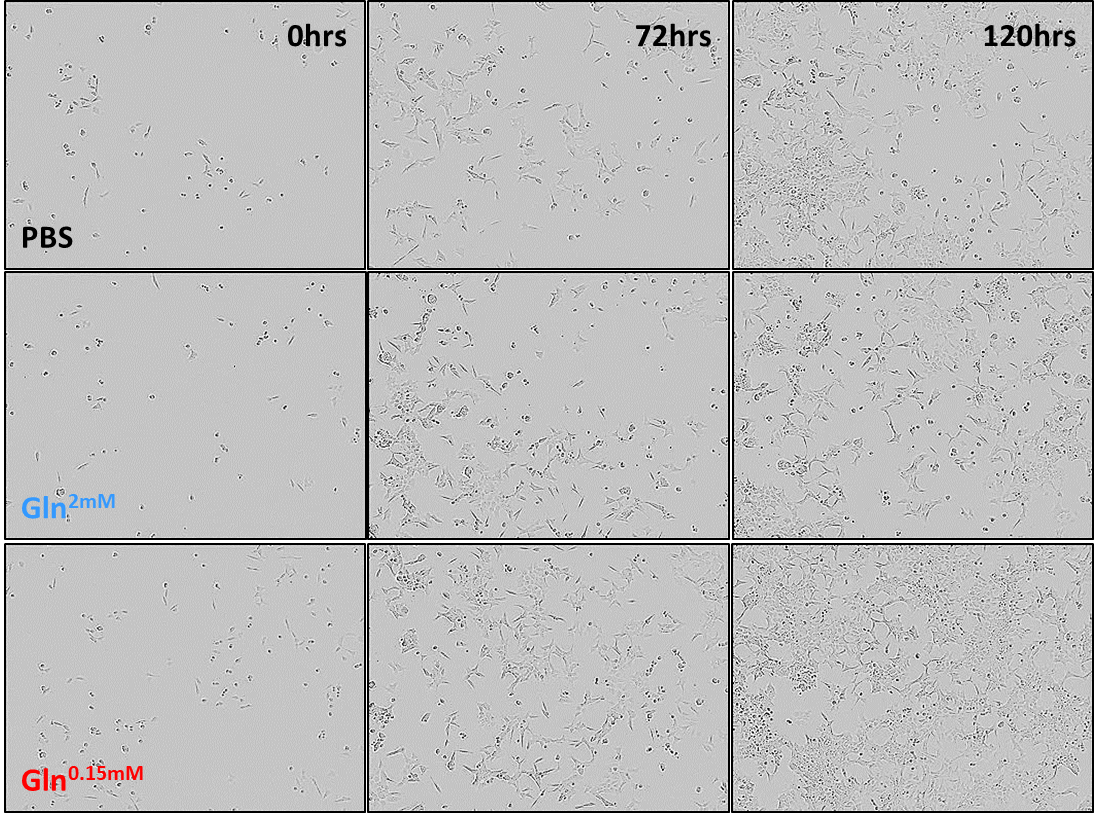
Fig. S4. Growth of HCT116 cells in the presence of low serum is stimulated by Rab11a-exosome-enriched sEV preparations.** Figure shows IncuCyte^®^ images of HCT116 cells grown following addition of PBS, or sEV preparations (4,000 sEVs/cell) from HCT116 cells secreted under normal glutamine (2 mM) and low glutamine (0.15 mM) conditions in the presence of 1% serum. sEVs secreted under low glutamine conditions contain increased levels of growth-promoting Rab11a-exosomes.


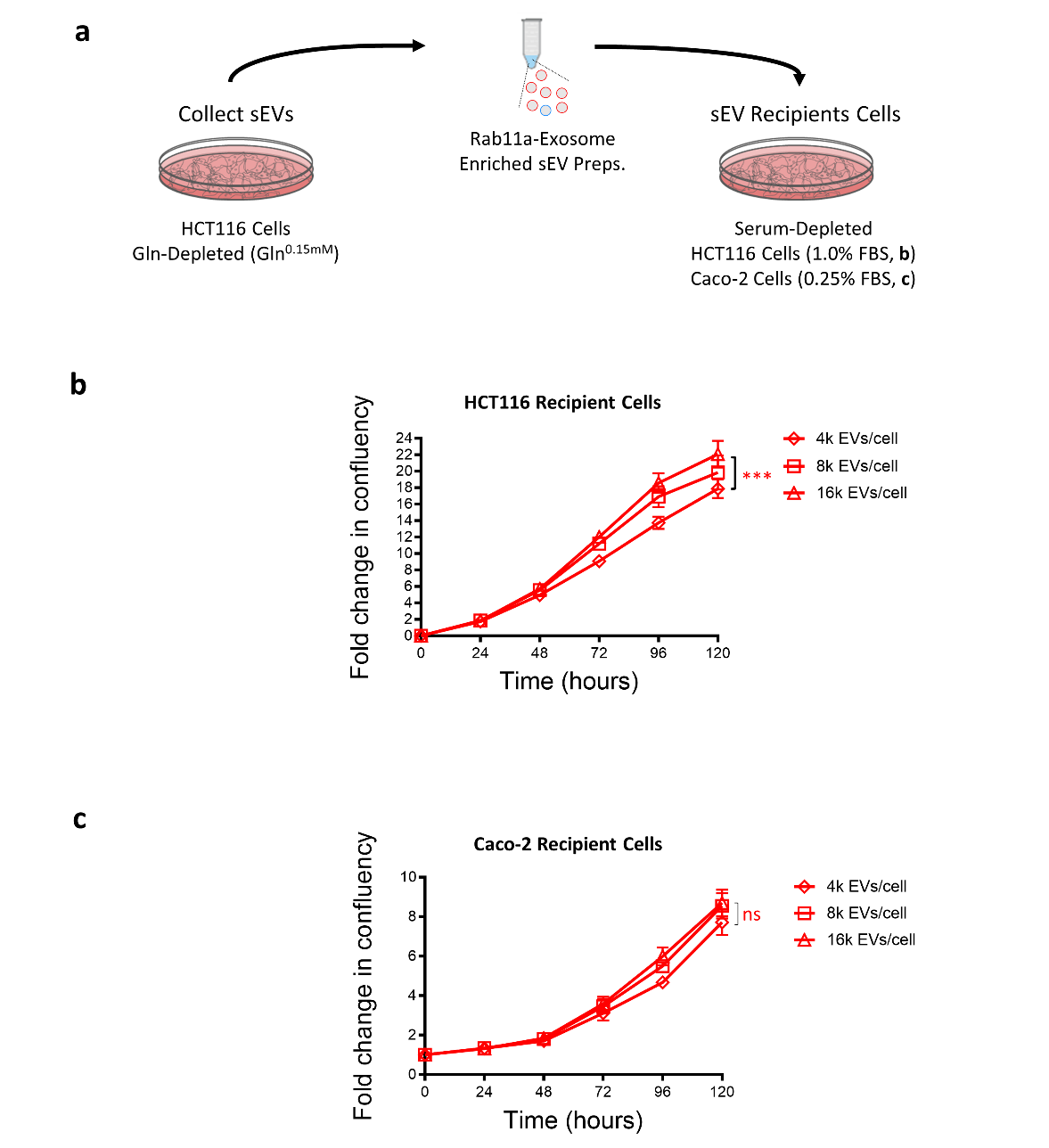


**Fig. S5.** **Rab11a-exosome preparations from HCT116 cells promote growth of HCT116 cells in a dose-dependent fashion under serum-depleted conditions.** (a) Experimental design relevant to the data below. (b) Growth-promoting activity of HCT116 Rab11a-exosome-enriched sEV preparations increases with dose for naïve HCT116 cells under 1% FBS conditions. (c) Growth-promoting activity of HCT116 Rab11a-exosome-enriched sEV preparations does not significantly increase with dose above 4000 sEVs per target cell for naïve Caco-2 cells under 0.25% FBS conditions. Eight technical repeats were employed for each condition. ***P<0.001.


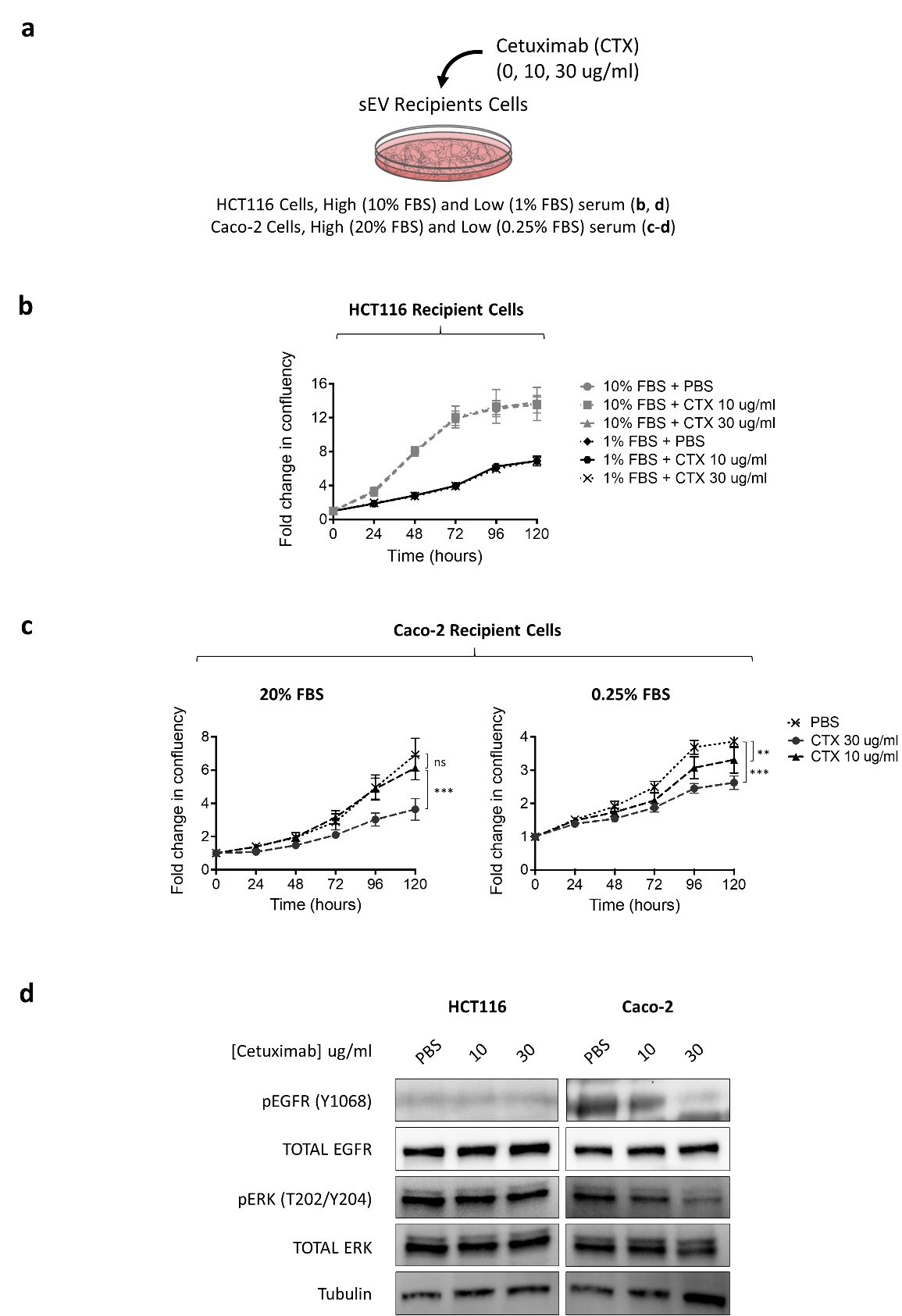


**Fig. S6. Cetuximab only reduces the growth of CRC cells carrying wild type KRAS****.** (a) Experimental design relevant to the data below. (b) Cetuximab at 10 and 30 µg/ml concentration has no effect on HCT116 cells grown under either high (10% FBS) or low (1%) serum conditions. Cetuximab resistance has previously been shown to involve the activated KRAS mutation in CRC cells. (c) Cetuximab inhibits growth of Caco-2 cells, which possess wild type KRAS, under high (20%) and low (0.25%) serum conditions in a dose-dependent manner. (d) Western blot of cell lysates showing that cetuximab treatment has no effect on EGFR or ERK phosphorylation in HCT116 cells, but reduces the phosphorylation of both proteins in Caco-2 cells in a dose-dependent manner. In growth experiments, eight technical repeats were employed for each condition and each experiment was repeated three times. **P<0.01; ***P<0.001.

**
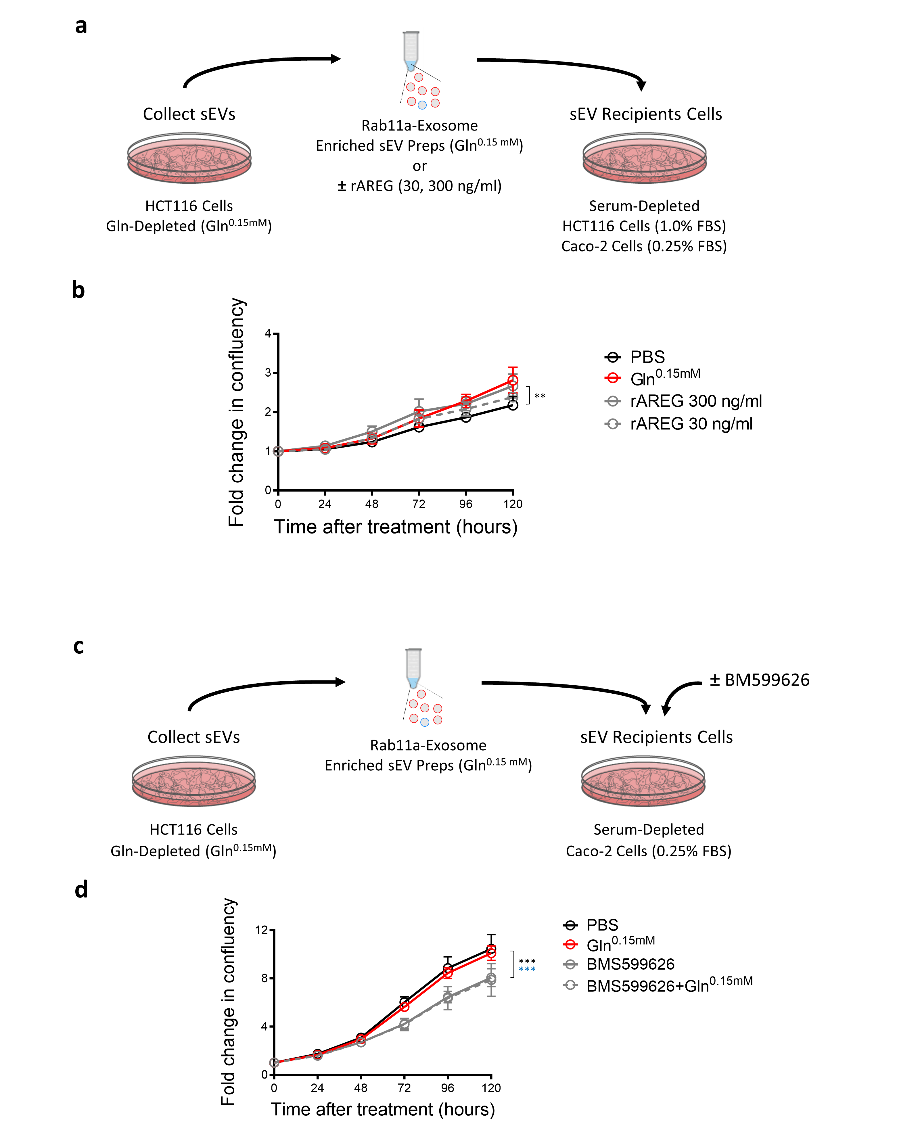
**

**Fig. S7. Further characterisation of the growth promoting effects of AREG on Rab11-exosomes.** (a) Experimental design relevant to (b). (b) Comparison of the growth-promoting effects on HCT116 cell growth over a 120-hour time course using soluble recombinant AREG (rAREG) and AREG on Rab11a-exosome-enriched sEV preparations (Gln^0.15 mM^). Black asterisks denote significant difference between 300 ng/ml rAREG-induced growth and control. (c) Experimental design relevant to (d). Schematic of experimental approach employed used to test the effect of BMS599626. (d) Comparison of the effect on Caco-2 cell growth over a 120-hour time course of Rab11a-exosome-enriched sEV preparations and PBS control in the absence and presence of pan-HER receptor tyrosine kinase inhibitor BMS599626 inhibitor. This experiment was repeated twice. For growth curves, eight technical repeats were employed for each condition. **P<0.01; ***P<0.001. In (d), black and blue asterisks denote significant inhibitory effects on PBS control and Rab11a-exosome-treated cells respectively.
